# Meta-analysis of genome-wide association studies of gestational duration and spontaneous preterm birth identifies new maternal risk loci

**DOI:** 10.1371/journal.pgen.1010982

**Published:** 2023-10-23

**Authors:** Anu Pasanen, Minna K. Karjalainen, FinnGen, Ge Zhang, Heli Tiensuu, Antti M. Haapalainen, Marja Ojaniemi, Bjarke Feenstra, Bo Jacobsson, Aarno Palotie, Hannele Laivuori, Louis J. Muglia, Mika Rämet, Mikko Hallman

**Affiliations:** 1 Research Unit of Clinical Medicine, Medical Research Center Oulu, University of Oulu, and Department of Children and Adolescents, Oulu University Hospital, Oulu, Finland; 2 Research Unit of Population Health, Faculty of Medicine, University of Oulu, Oulu, Finland; 3 Division of Human Genetics, Cincinnati Children’s Hospital Medical Center, Center for Prevention of Preterm Birth, Perinatal Institute and March of Dimes Prematurity Research Center Ohio Collaborative, Cincinnati Children’s Hospital Medical Center, Department of Pediatrics, University of Cincinnati College of Medicine, Cincinnati, Ohio, United States of America; 4 Department of Epidemiology Research, Statens Serum Institut, Copenhagen, Denmark; 5 Department of Obstetrics and Gynaecology, Sahlgrenska Academy, Institute of Clinical Science, University of Gothenburg, Gothenburg, Sweden; 6 Department of Genetics and Bioinformatics, Health Data and Digitalization, Norwegian Institute of Public Health, Oslo, Norway; 7 Institute for Molecular Medicine Finland (FIMM), Helsinki Institute of Life Science, University of Helsinki, Helsinki, Finland; 8 Program in Medical and Population Genetics, Broad Institute of Harvard and MIT, Cambridge, Massachusetts, United States of America; 9 Psychiatric & Neurodevelopmental Genetics Unit, Department of Psychiatry, Analytic and Translational Genetics Unit, Department of Medicine, and the Department of Neurology, Massachusetts General Hospital, Boston, Massachusetts, United States of America; 10 Center for Child, Adolescent, and Maternal Health Research, Faculty of Medicine and Health Technology, University of Tampere, Tampere, Finland; 11 Department of Obstetrics and Gynecology, Tampere University Hospital, Tampere, Finland; 12 Burroughs Wellcome Fund, Research Triangle Park, Durham, North Carolina, United States of America; 13 Faculty of Medicine and Health Technology, Tampere University, Tampere, Finland; University at Buffalo - The State University of New York, UNITED STATES

## Abstract

**Background:**

Preterm birth (<37 weeks of gestation) is a major cause of neonatal death and morbidity. Up to 40% of the variation in timing of birth results from genetic factors, mostly due to the maternal genome.

**Methods:**

We conducted a genome-wide meta-analysis of gestational duration and spontaneous preterm birth in 68,732 and 98,370 European mothers, respectively.

**Results:**

The meta-analysis detected 15 loci associated with gestational duration, and four loci associated with preterm birth. Seven of the associated loci were novel. The loci mapped to several biologically plausible genes, for example *HAND2* whose expression was previously shown to decrease during gestation, associated with gestational duration, and *GC* (Vitamin D-binding protein), associated with preterm birth. Downstream *in silico*-analysis suggested regulatory roles as underlying mechanisms for the associated loci. LD score regression found birth weight measures as the most strongly correlated traits, highlighting the unique nature of spontaneous preterm birth phenotype. Tissue expression and colocalization analysis revealed reproductive tissues and immune cell types as the most relevant sites of action.

**Conclusion:**

We report novel genetic risk loci that associate with preterm birth or gestational duration, and reproduce findings from previous genome-wide association studies. Altogether, our findings provide new insight into the genetic background of preterm birth. Better characterization of the causal genetic mechanisms will be important to public health as it could suggest new strategies to treat and prevent preterm birth.

## Author summary

Annually, more than 15 million pregnancies are affected by preterm births all over the world. There are no effective ways to prevent preterm birth, and premature babies suffer from neonatal mortality and lifelong morbidities. Genetic factors of mother and fetus explain a large proportion, approximately 30–40%, of the of the variation in gestational age at delivery. To date, there have been just a few unbiased genome-wide investigations set out to locate these genes. Better characterization of the causal genetic mechanisms could suggest new strategies to treat and prevent preterm birth. In the current study, we aimed to identify maternal genetic factors that contribute to the timing of birth by meta-analyzing genome-wide association studies from European populations. We detected 17 independent loci that were associated with gestational duration and/or the risk of preterm birth. Ten of the loci replicated associations from previous studies, and seven were novel. The replicated associations provide strong evidence for the importance of these loci in the timing of birth, although the exact genes and causal variants and pathways still require further functional analysis. The seven novel associations provide further intriguing candidates that may account for the risk of preterm birth. Bioinformatics analysis proposed the associated loci have regulatory functions predominantly in immune cell types and reproductive tissues. The analysis further highlighted the unique nature or preterm birth as a phenotype, since the only traits with strong correlations were birth weight measures that are closely linked to the studied phenotypes. Our findings complement the knowledge of the genetic factors of preterm birth.

## Introduction

Proper timing of birth is crucial for the survival and long-term health of newborn infants. Preterm birth, defined as birth that occurs prior to 37 completed weeks of gestation, is the most common cause of neonatal death and a prevalent cause of death among children under 5 years [1]. Moreover, preterm birth is the underlying cause of several long-term morbidities including neurodevelopmental problems, cerebral palsy, learning difficulties, and sensory loss [2]. Globally, preterm birth affects approximately 11% of births, equal to 15 million pregnancies, each year. In Scandinavian countries and Finland, the annual incidence of preterm birth is approximately 5–6% [2, 3].

While intrauterine growth restriction and preeclampsia are the major causes of medically indicated preterm birth, approximately 70% preterm births occur after spontaneous onset of labor [1]. There are just a few ways to predict the risk [4] and no efficient ways prevent the occurrence of spontaneous preterm birth (SPTB). Genetic variants in maternal and fetal genomes have been recognized as factors that contribute to the risk of SPTB and to variation in gestational duration. Family studies suggest that approximately 30%–40% of the variation in timing of birth is explained by genetic factors, with contributions from the maternal genome most important [5–7]. Recent genome-wide association studies (GWAS) have identified some robust associations. Variants in genes including *WNT4*, *EBF1*, *AGTR2*, and *KCNAB1* were associated with timing of birth in mothers [8, 9], and a study with fetal samples discovered a locus near genes that encode pro-inflammatory cytokines associated with gestational duration [10].

In the present study, our aim was to strengthen knowledge of the genetic background of SPTB by identifying and replicating associations of genetic loci in relation to timing of spontaneous singleton birth. To that end, we conducted a case-control meta-analysis of SPTB and a quantitative meta-analysis of gestational duration in 98,370 and 68,732 European mothers, respectively.

## Results

### Overview of genome-wide meta-analysis

**Fig 1:**
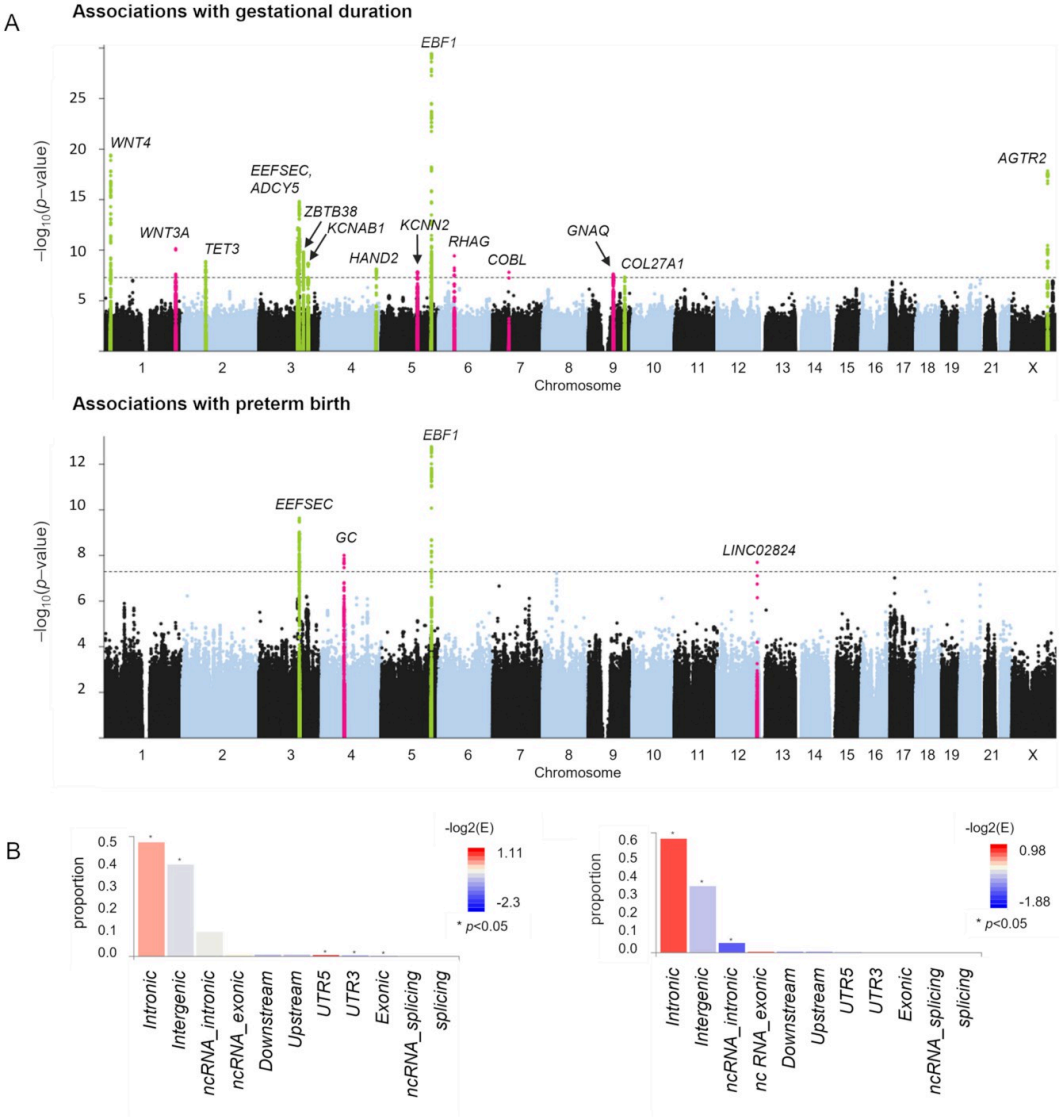
Meta-analysis of gestational duration and SPTB. A: Loci with genome-wide significant associations (*p*<5×10^–8^) are highlighted in the Manhattan plots. Chromosomal positions are shown at the x-axis, and the y-axis shows association *p* values at the –log_10_ scale. The meta-analysis detected 15 loci associated with gestational duration and four loci associated with SPTB. Peaks highlighted in pink represent novel loci, and peaks highlighted in green show known loci B: Annotations as number of SNPs per functional consequences on genes in the analysis of gestational duration (on the left) and preterm birth (on the right). Bars are colored by –log_2_(enrichment) relative to all variants in the reference panel.

The meta-analysis of SPTB (*n* = 98,370) and gestational duration (*n* = 68,732) was conducted with maternal GWAS data from the FinnGen study, 23andMe, Inc., and the cohort from Northern and Central Finland S1 Fig. 84,1% of the women delivered at term (37–42 weeks), 11,4% delivered preterm (<37 weeks), and 4,4% had post term births (>42 weeks) (S2 Table). We detected 17 independent loci at least 1Mb apart and with at least one variant associated at *p*<5×10^–8^ (Fig 1A and Tables 1 and 2). Fifteen loci were associated with gestational duration, and four with SPTB. The loci near *EBF1* and *EEFSEC* were associated with both gestational duration and SPTB, as also shown in previous GWAS and meta-analysis with maternal data [8, 9]. The associated variants were mostly annotated as intronic or intergenic, and the associations for gestational duration were also nominally enriched for UTR-regions and exones (Fig 1B. We considered an associated locus to be novel if there were no genome-wide significant associations with gestational duration or SPTB for any of the variants within a ±1 Mb range around the meta-analysis lead variant in the GWAS Catalog [11] (S3 Table) or around the loci detected in a recent maternal meta-analysis of the timing of parturition [9]. We detected five novel loci associated with gestational duration, and two novel risk loci for SPTB. The results of the meta-analysis with a strict definition for spontaneous birth in the FinnGen GWAS are shown in S2 Fig. The effect estimates of the associated loci were similar among the FinnGen-based analysis of SPTB and GWAS with the strict definition for spontaneous birth S3 Fig.

**Table 1:** Loci associated with gestational duration in meta-analysis of 68,732 women of European ancestry. Loci highlighted in bold had no previous associations (*p*<5×10^-8^) with gestational duration or preterm birth.

Chr:Pos	Rsid	A1	Freq	Z-score	*p* -value	HetPVal	Gene
5: 158460958	rs6881817	T	0.244	-11.41	3.74×10^-30^	0.726	*EBF1*
1: 22141722	rs3820282	T	0.149	9.189	3.95×10^-20^	0.075	*WNT4*
23: 116053119	rs5950512	A	0.401	-8.791	1.48×10^-18^	0.512	*AGTR2*
3: 128159974	rs2999049	T	0.720	-7.972	1.56×10^-15^	0.258	*EEFSEC*
3: 123365694	rs10934646	A	0.634	-7.193	6.35×10^-13^	0.121	*ADCY5*
**1:228015567**	**rs708119**	**C**	**0.670**	**6.514**	**7.32×10^-11^**	**0.207**	***WNT3A***
3: 141414608	rs1991431	A	0.441	6.402	1.54×10^-10^	0.583	*ZBTB38*
**6:49592080**	**rs10948514**	**T**	**0.274**	**6.267**	**3.68×10^-10^**	**0.999**	***RHAG***
2: 74009288	rs71848031	D	0.057	-6.061	1.36×10^-9^	0.913	*TET3*
3: 156137629	rs4679760	C	0.426	-5.992	2.07×10^-9^	0.360	*KCNAB1*
4: 173813320	rs7697038	A	0.323	5.780	7.45×10^-9^	0.355	*HAND2*
**5:114208039**	**rs13175113**	**T**	**0.486**	**5.668**	**1.45×10^-8^**	**0.174**	***KCNN2***
**7:51940143**	**rs151143987**	**T**	**0.996**	**5.655**	**1.56×10^-8^**	**0.712**	***COBL***
**9:77936015**	**rs11145617**	**A**	**0.210**	**-5.586**	**2.32×10^-8^**	**0.584**	***GNAQ***
9: 114160401	rs2808791	T	0.474	5.467	4.58×10–8	0.429	*COL27A1*

Chr, chromosome; Pos, position (Grch38); A1, effect allele; HetPval, *p* value for heterogeneity. Novel loci are highlighted in bold; reported genes are the nearest genes. For replicated loci, candidate genes are based on previous studies.

**Table 2:** Loci associated with SPTB in meta-analysis of 98,370 women of European ancestry. Loci highlighted in bold had no previous associations (*p*<5×10^-8^) with gestational duration or preterm birth.

Chr:Pos	Rsid	A1	Freq	OR	CI95%	*p*-value	HetPVal	Gene
5: 158467739	rs12520982	T	0.714	0.818	0.78–0.86	1.71×10^-13^	0.385	*EBF1*
3: 128348271	rs1553758423	D	0.707	1.210	1.14–1.28	2.32×10^-10^	0.630	*EEFSEC*
**4:71763252**	**rs2276461**	**A**	**0.052**	**1.290**	**1.18–1.41**	**9.84×10^-9^**	**0.626**	***GC***
**12:126705821**	**rs192808132**	**A**	**0.993**	**0.581**	**0.48–0.70**	**1.99×10^-8^**	**0.462**	***LINC02824***

Chr, chromosome; Pos, position (Grch38); A1, effect allele; OR, odds ratio; CI95%, 95% confidence interval; HetPval, *p* value for heterogeneity. Novel loci are highlighted in bold; reported genes are the nearest genes. For replicated loci, candidate genes are based on previous studies.

**Fig 2:**
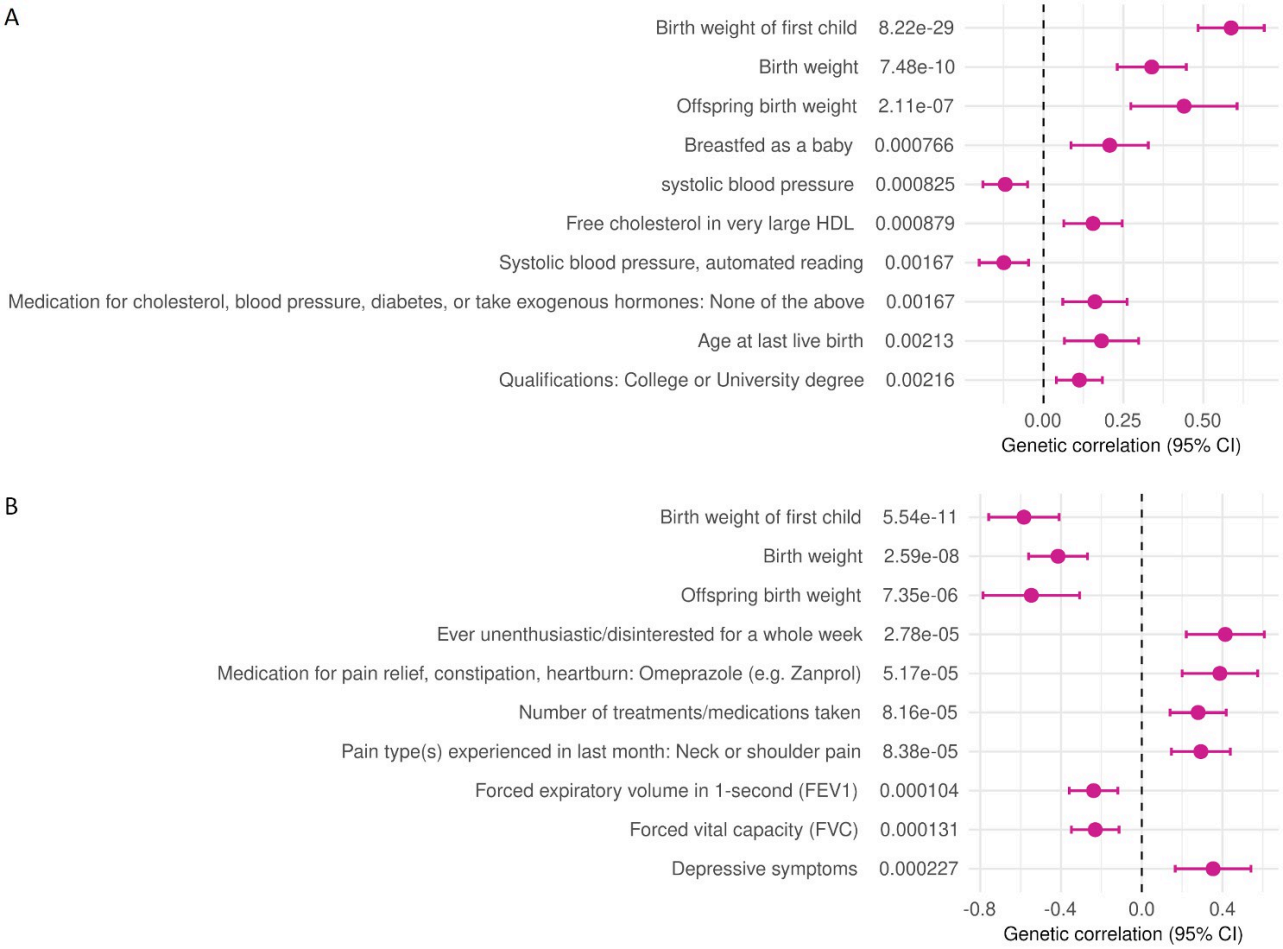
Genetic correlations between A) gestational duration or B) SPTB and other complex traits. Genetic correlation between gestational duration or SPTB and a comprehensive set of 773 complex traits was analyzed with LD score regression. Top 10 correlated traits, followed by their respective *p* values, are shown.

Linkage disequilibrium score regression (LDSC)-based genomic inflation factor [12] indicated minimal confounding effects in the meta-analysis of gestational duration (λGC = 1.077, intercept = 1.025) or SPTB (λGC = 1.038, intercept = 1.007) S4 Fig. The meta-analysis test statistics were homogenous among populations, and the effect estimates of the risk loci for SPTB were similar across individual cohorts (Tables 1 and 2 and S5 Fig). According to LDSC (SNP)-based heritability estimates, the current results explain approximately 17.5% of the variation in gestational duration and 6% of SPTB on a liability scale (S4 Table). We further used LDSC to evaluate shared genetic architecture between the meta-analysis outcomes and 773 other complex traits (Fig 2 and S5 Table). The analysis detected significant correlations between birth weight–related measures and both gestational duration and SPTB. As expected, longer duration of gestation was associated with higher birth weight, whereas preterm birth was linked to lower birth-weight measures. In addition, specific measures of physical fitness, alertness, and lack of depression correlated with a longer duration of pregnancy or term birth.

MAGMA gene set enrichment analysis based on the full distribution *p*-values indicated involvement of gonad development and steroid hormone biosynthesis in gestational duration, whereas kinetochore-microtubule and neuron differentiation were the top pathways in SPTB (S6 Table). MAGMA tissue expression analysis across GTEx v8 did not yield significant results but ranked several reproductive tissues, including uterus and ovary, among the most relevant tissue types for both gestational duration- and SPTB-associated genes (S6 Fig). When visualized in a gene-expression heatmap across the GTEx v8 tissues, some of the genes, including *HAND2*, *ZBTB38*, *GNAQ*, and *COL27A1*, clustered in a profile of higher expression in blood vessel and in female reproductive tissues such as uterus, cervix uteri, and fallopian tube (S6 Fig). Regional association plots for the novel loci for gestational duration are shown in S7 Fig and for SPTB in S8 Fig.

### Replication and joint analysis

We used data from the Nordic data sets to test for replication of the associated loci and to perform joint analysis (S7 Table). Loci near *WNT4*, *EEFSEC*, *EBF1*, and *AGTR2* were not included, since the same replication data was used in the study that discovered these associations, and our meta-analysis also replicated these associations [8]. While all effects among the genome-wide significant meta-analysis loci and the replication population were in the same direction, the strongest associations in the replication population were detected for *ZBTB38*, *HAND2*, *TET3*, and *KCNAB1*. These loci were also associated in a recent meta-analysis of gestational duration [9]. Joint analysis of the replication data and the meta-analysis variants with suggestive significance (*p*<1×10^-6^ to 5×10^-8^) detected *DNAH2* and *RAP2C* as additional loci associated with gestational duration. The association for the *RAP2C* locus was previously known [8], while the association for the *DNAH2* was novel. Gene set analysis based on a list of genes corresponding to all significant loci in the current study identified enrichment of multiple pathways, with GO terms referring to regulation of morphogenesis and development of various organs and tissues among the top pathways (S8 Table).

### Characterization of association signals

To gain insight into the nature of the associated loci, we explored previous associations with other complex traits in the literature and with data from FinnGenR7 and the IEU open GWAS project [13], and performed colocalization analysis with expression quantitative trait loci (eQTLs) to evaluate if the associated variants affect their target genes by regulating gene expression. We report loci with posterior probability of colocalization (PP)>0.6. In the FinnGen data, we screened the meta-analysis lead variants for associations with all >3000 phenotypes in freeze 7 (S9 Fig), whereas data from the IEU openGWAS project was queried in a PheWAS for all associated variants within the associated meta-analysis loci (S9 Table).

Loci near *EBF1*, *EEFSEC*, *WNT4*, *ADCY5*, and *AGTR2* were associated with gestational duration or SPTB in two previous genome-wide investigations with maternal data [8, 9], and the current meta-analysis replicated those associations. These loci will not be reviewed. We detected replicable associations for *ZBTB38*, *HAND2*, *TET3*, and *KCNAB1* with gestational duration, also associated in another recent meta-analysis of the timing of birth [9]. Colocalization analysis provided evidence that gene regulation of the loci near *WNT3A* (novel), *ADCY5*, and *KCNAB1* could play a role of regulating pregnancy duration in reproductive tissues, and further suggested that many previously known (*EEFSEC*, *ZBTB38*, *EBF1*, *COL27A1*, *HAND2*, *TET3*) or novel (*GNAQ*) loci have regulatory roles in immune cell types (S10 Table). In addition, we detected three novel loci associated with gestational duration (*RHAG*, *KCNN2*, *COBL*) in the meta-analysis, and one novel locus (*DNAH2*) in the joint analysis of the meta-analysis and replication data, for which the genes were assigned based on proximity in the lack of previous association with SPTB or evidence for colocalization. The case-control meta-analysis of SPTB replicated the associations for *EBF1* and *EEFSEC*, and detected two novel associated loci (*GC*, *LINC02824*), for which the genes were assigned based on proximity.

**Fig 3:**
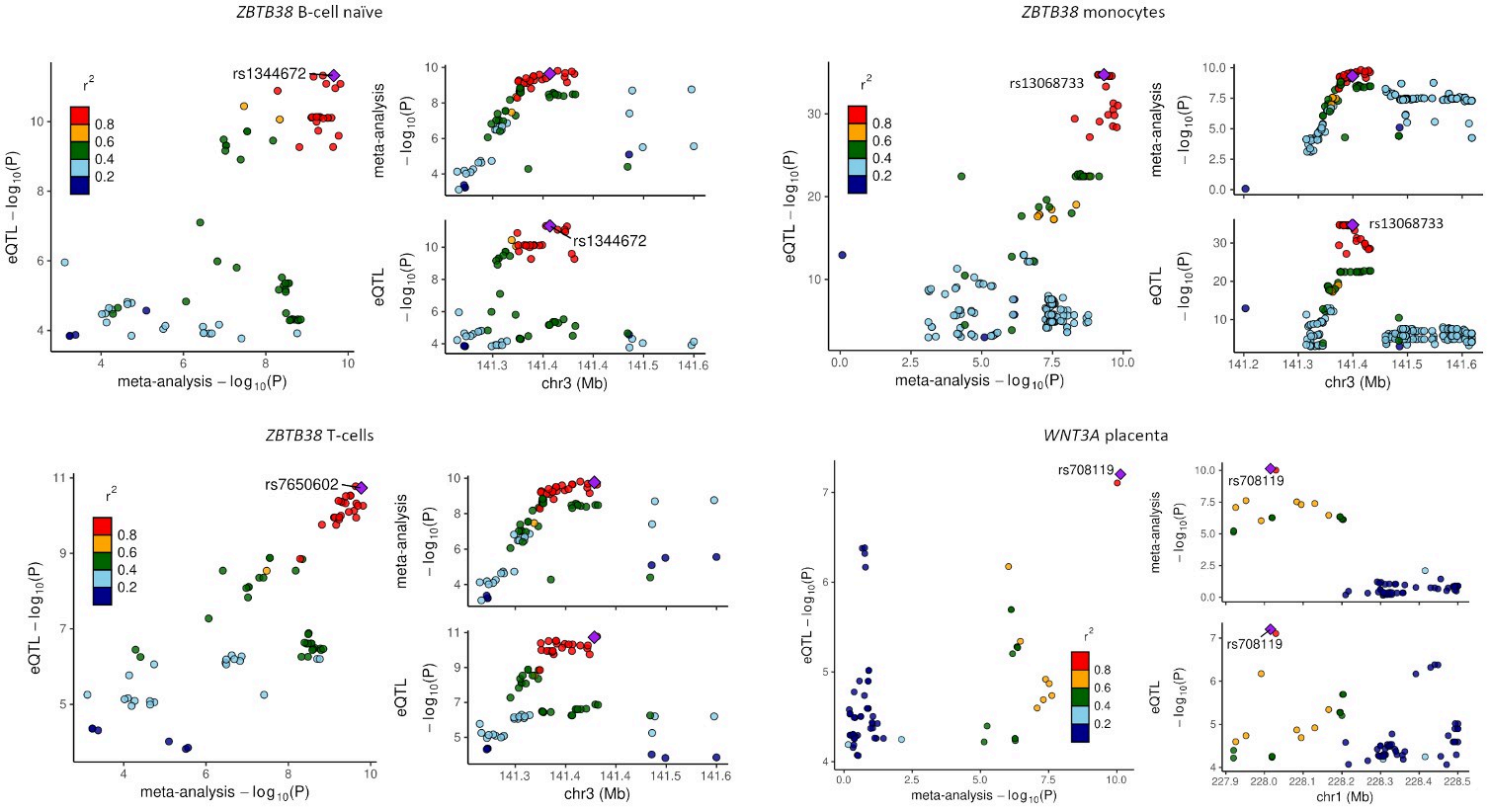
Colocalization analysis of meta-analysis associations with expression quantitative trait (eQTL) data. The variants are colored according to their LD (r^2^) with the lead SNP, based on pairwise LD in European population of the 1000 Genomes Project Phase 3. A–C) *ZBTB38* variants were implicated in gestational duration–linked gene regulation in various cell types including naïve B cells (PP = 0.95), monocytes (PP = 0.90), and T cells (PP = 0.95). D) Variants in gestational duration–associated *WNT3A* locus showed strongest colocalization with *WNT3A* expression in placenta (PP = 1.00).

The lead variant rs1991431 in *ZBTB38* with a replicable association was associated with hyperplasia of prostate (BHP) in the FinnGen (S9 Fig), and other associated variants in the locus were linked to various complex traits including cell counts of lymphocytes and monocytes, and *ZBTB38* mRNA expression on the IEU openGWAS data. The same alleles of several meta-analysis variants (e.g., T allele of variant rs9846396 associated with longer gestational duration; Z-score = 6.36, *p* = 2.04×10^-10^) were also associated with taller height, higher body mass measures, and increased risk of prostate cancer (S9 Table). We detected colocalization for variants associated with gestational duration and *ZBTB38* expression in tissues including monocytes, T cells, and B cells, and alleles associated with longer gestational duration were linked to higher *ZBTB38* expression (Fig 3 and S10 Table).

We detected a replicable association of variants near *HAND*. This gene plays a role in cardiac development with previous associations with traits including atrial fibrillation and platelet count [11]. *HAND2* is expressed in the human uterine tissue, where it is upregulated by the progesterone receptor, and involved in immune tolerance of the decidua by regulating a distinct set of genes, including interleukin 15 [14, 15].

Variants in *TET3* and *KCNAB1* with replicable associations were associated with birth weight of the offspring (S9 Table), and alleles associated with longer gestational duration in the current meta-analysis were linked to higher birth-weight measures. We observed a similar positive correlation between gestational duration and birth weight at the genome-wide level in the LDSC analysis (Fig 2). *KCNA1B*-variants associated with gestational duration colocalized with *KCNA1B* expression in two reproductive tissues and in blood vessel (S10 Table). Alleles linked to longer gestational duration were associated with increased *KCNAB1* expression in all tissues.

Variants in *COL27A1* showed previous associations with traits including sex-hormone binding globulin measurement, birth weight, and blood cell type proportions (S3 Table). Gestational duration-associated variants colocalized with *COL27A1* expression in lymphoblastoid cells (LCLs) (S10 Table). The alleles associated with longer gestational duration were linked to decreased *COL27A1* expression.

Like variants in *ZBTB38*, the polymorphisms in the *WNT3A* locus were associated with height and body mass indices, and the encoded protein was implicated in cell fate and patterning during embryogenesis [16, 17]. Concordantly, variants associated with gestational duration colocalized with *WNT3A* expression in the placenta (Fig 3 and S10 Table). The placental tissues were collected from the fetal membrane side [18]. We further investigated the gene-expression patterns of *WNT3A* in publicly available single cell data with placental tissues from human pregnancies and found evidence for the localization of *WNT3A* expression in fetal fibroblasts [19–21] and placental smooth muscle cells [22]. Further, *WNT3A* was enriched for trophoblast ligand-receptor interaction with *SFRP2* in cytotrophoblast cell column [21].

Variants in the *GNAQ* locus were previously associated with body mass index, hemoglobin measurements and cell type properties of erythrocytes and reticulocytes (S3 Table), and colocalization analysis provided some evidence for pregnancy-related regulation of *GNAQ* in monocytes S10 Table). *GNAQ* was identified as part of a transcriptomic signature related to human labor in the choriodecidua, and according to a human cell atlas of fetal development 16% of placental cells express *GNAQ* [22, 23]. Trophoblasts collected during the first trimester of pregnancy from maternal and fetal side also expressed *GNAQ* [21].

The case-control meta-analysis of SPTB detected two novel associated loci: *GC* and *LINC02824*, which encode vitamin D-binding protein and a long noncoding RNA, respectively. *GC* is involved in vitamin D transport and storage, and circulating vitamin D levels have been linked to preterm birth and other pregnancy- and reproductive health–related outcomes in observational studies [24, 25].

## Discussion

The current genome-wide meta-analysis of SPTB (*n* = 98,370) and gestational duration (*n* = 68,732) identified several associated loci. We detected loci that had no previous associations with gestational duration or SPTB, and our findings further reinforce the associations of genes from previous GWASs of mothers who gave birth preterm. The associated loci with strong replication in the current analysis included *ZBTB38*, *HAND2*, *TET3*, and *KCNAB1*. These loci, along with *COL27A1*, also showed association in the recent meta-analysis of the timing of parturition [9], establishing these genes as strong candidates for further molecular biological studies of SPTB. The inferred functions of the assigned candidate genes were consistent with a role in the timing of birth.

Association of *ZBTB38*, zinc finger and BTB domain containing 38, with benign hyperplasia of prostate (BHP) is a compelling finding given that both gestational duration and BHP are affected by changes in estrogen and androgen levels [26, 27]. *ZBTB38* was further associated with cell counts of various immune cells, and our results suggest that increased *ZBTB38* expression in these cell types may play a role in regulating length of pregnancy. Alleles associated with longer gestational duration showed association with increased height, body mass, and risk of prostate cancer. It remains to be determined whether *ZBTB38* confers its effect on birth timing through pregnancy-specific mechanisms or by contributing to more general immune pathways that influence gestation. Our findings for *ZBTB38* associations are in keeping with reported correlations among maternal height, gestational duration, and fetal growth, and further comply with detected associations between maternal birth weight–elevating alleles and longer gestational duration and between maternal gestation-prolonging alleles and the risk of prostate carcinoma [8, 28, 29].

An association near *HAND2* showed strong replication. *HAND2* encodes heart and neural crest derivatives expressed 2, a transcription factor best known for its roles in cardiac morphogenesis and limb development. Decreasing expression of *HAND2* in the decidua during pregnancy may contribute to regulation of gestational duration [30]. The expression of *HAND2* in the human uterine tissue, and its gradually decreasing expression in the decidua during pregnancy [30], makes it an interesting candidate gene and a potential biomarker for SPTB.

Variants in Tet methylcytosine dioxygenase 3 (*TET3*) and potassium voltage-gated channel subfamily A regulatory beta subunit 1 (*KCNAB1*) were previously associated with birth weight of offspring, and alleles associated with longer gestational duration correlated with birth-weight measures, complying with known correlations of the length of gestation and fetal growth [28]. It is possible that the associations between these loci and gestational duration explain the effect of the mentioned loci on birth weight. Our results further suggest that *KCNAB1* expression contributes to the timing of birth. Of note, *TET3* was suggested to play a role in embryo implantation [31]. Both *TET3* and *KCNAB1* represent interesting targets for further study to determine their specific roles related to the regulation of gestational duration.

Interestingly, *COL27A1* was associated with phenotypes including embryonic growth retardation, abnormal placenta morphology, and abnormal placenta vasculature in data in the knock-out mice as investigated via IMPC (https://www.mousephenotype.org/). *COL27A1* is most abundantly expressed in the endometrium, and the gene encodes collagen type XXVII alpha 1 chain, which is a fibrillar, developmentally regulated protein. Further, the meta-analysis lead variant of the *COL27A1* locus is near miR-455, which has roles in cartilage development, adipogenesis, and preeclampsia, and may protect endometrial cells against oxidative stress [32, 33].

The novel meta-analysis loci associated with gestational duration, comprised further intriguing candidates. For the loci near *WNT3A* and *GNAQ*, these genes were also supported as causal genes in the colocalization analysis. The genes further had some known functions consistent with a role in the timing of birth. Our results and previous assessments with single cell data suggest that *WNT3A* could play a role in regulation of pregnancy in both maternal and fetal tissues of the maternal-fetal interface during pregnancy [19–21]. *GNAQ* (protein subunit alpha q) plays a role in survival of immune cells. Interestingly, it occurs as a part of a transcriptomic signature related to human labor in the choriodecidua [23, 24, 34]. Moreover, *GNAQ* is widely expressed in cells of the maternal-fetal interface [20, 21].

The novel locus near *KCNN2*, associated with gestational duration, had some roles that could link to regulation of pregnancy length. *KCNN2* encodes a potassium calcium-activated channel subfamily N member 2, and potassium channel proteins have been linked to uterine function during gestation [35]. We found no obvious connection of *KCNN2* to pregnancy-related regulation, but *KCNN3*, another molecule in the *KCNN* family of potassium channel genes, plays a role in uterine function [36]. The associations for *KCNN2* and other novel risk loci should be replicated in independent populations, and the role of the risk loci and corresponding candidate genes remains to be verified.

The case-control meta-analysis of SPTB detected an association in *GC*, encoding GC vitamin D binding protein, as of special interest because of its known involvement in vitamin D transport and storage. Protein encoded by *GC* is the primary carrier of vitamin D that binds to the vitamin and its plasma metabolites and transports them to their target tissues. Previous studies have suggested links among plasma vitamin D levels and preterm birth or other pregnancy- related outcomes including pre-eclampsia, polycystic ovary syndrome, and endometriosis [24, 25]. Vitamin D deficiency was found associated with many adverse outcomes including those related to pregnancy, whereas increased levels of the protein product of *GC* showed association with a reduced risk of certain immune-mediated diseases [25, 37, 38]. The precise role of *GC* in the context of human pregnancy and SPTB remains to be determined.

Altogether, our results highlight the unique nature of SPTB. At the genome-wide level, birth weight measures were the only traits that showed significant correlations with both gestational duration and SPTB in a comprehensive set of complex phenotypes. However, the Bonferroni correction deployed for the 773 tests is likely overly conservative since the traits include closely related phenotypes. Gene set enrichment for gestational duration and pathway analysis for gestational duration and SPTB highlighted involvement of gonad development and steroid hormone biosynthetic processes and GO terms referring to regulation of morphogenesis among multiple top pathways. Gene set enrichment analysis of SPTB implied kinetochore microtubule as the top pathway. Proper function of the kinetochore-microtubule pathway is essential for preserving genomic integrity and prevention of birth defects [39]. Tissue analysis pinpointed several reproductive tissues, including uterus and ovary, among the most relevant tissue types for both SPTB- and gestational duration-associated genes.

Multiple variants in the candidate loci were individually associated with birth weight indices. Associated genes had primary roles in steroid hormone–regulating processes and tissue and organ morphogenesis. Reproductive tissues of the mother were among the principal locations where the associated genes were expressed. Our results suggest that many of the associated variants contribute to pregnancy outcomes by regulating expression of their target genes, mainly but not exclusively in reproductive tissues and immune cell types. Hence, our results indicate that those tissue and cell types are the most relevant when considering the regulatory events related to pregnancy and preterm birth, and should be the primary targets in future molecular biological studies of SPTB and gestational duration.

The current analysis was restricted to individuals of predominantly European descent. Future studies should include ancestrally diverse populations to better understand the genetic architecture of the timing of birth and to ensure the broad applicability of results from genetic studies [40].

In conclusion, the current meta-analysis detected multiple loci that were associated with gestational duration or SPTB and produced intriguing candidates for further studies. Our results highlight the intricate nature of spontaneous birth as a trait and emphasize the importance of reproductive and immune tissues and cell types. The new genetic discoveries prime further research including large-scale complex investigations and individual regulatory pathway analyses utilizing labor-inducing tissues and cells. Studies may eventually reveal signaling pathways that activate spontaneous preterm birth and contribute towards effective prevention of SPTB.

## Methods

We conducted a meta-analysis of SPTB (8,542 cases and 89,828 controls) and a quantitative meta-analysis of gestational duration (*n* = 68,732) with data from mothers of European ancestry. The data originated from FinnGen, Northern/Central Finland, and 23andMe project.

### Ethics statement

FinnGen participants provided informed consent under the Finnish Biobank Act. Older cohorts with study-specific consents were transferred to the Finnish biobanks after approval by Fimea, the National Supervisory Authority for Welfare and Health. Recruitment protocols followed the biobank protocols approved by Fimea. The Coordinating Ethics Committee of the Hospital District of Helsinki and Uusimaa (HUS) approved the FinnGen study protocol (Nr HUS/990/2017). The FinnGen study is approved by the Finnish Institute for Health and Welfare (permit numbers THL/2031/6.02.00/2017, THL/1101/5.05.00/2017, THL/341/6.02.00/2018, THL/2222/6.02.00/2018, THL/283/6.02.00/2019, THL/1721/5.05.00/2019, THL/1524/5.05.00/2020, and THL/2364/14.02/2020), the Digital and Population Data Service Agency (permit numbers VRK43431/2017-3, VRK/6909/2018-3, and VRK/4415/2019-3), the Social Insurance Institution (permit numbers KELA 58/522/2017, KELA 131/522/2018, KELA 70/522/2019, KELA 98/522/2019, KELA 138/522/2019, KELA 2/522/2020, and KELA 16/522/2020), and Statistics Finland (permit numbers TK-53-1041-17 and TK-53-90-20). The Biobank Access Decisions for FinnGen samples and data utilized in FinnGen Data Freeze 6 include: THL Biobank BB2017_55, BB2017_111, BB2018_19, BB_2018_34, BB_2018_67, BB2018_71, BB2019_7, BB2019_8, BB2019_26, BB2020_1, Finnish Red Cross Blood Service Biobank 7.12.2017, Helsinki Biobank HUS/359/2017, Auria Biobank AB17-5154, Biobank Borealis of Northern Finland_2017_1013, Biobank of Eastern Finland 1186/2018, Finnish Clinical Biobank Tampere MH0004, Central Finland Biobank 1-2017, and Terveystalo Biobank STB 2018001. Women from Northern/Central Finland provided written informed consent, and studies were approved by ethics committee of Oulu University Hospital (79/2003, 14/2010, and 73/2013). Women from the 23andMe provided written informed consent and completed online surveys according to a human-subjects protocol approved by Ethical and Independent Review Services (www.eandireview.com).

### Study cohorts and phenotype descriptions

The summary of the phenotype and genotype data processing of the meta-analysis populations is shown in S1 Table.

**The FinnGen research project** (launched 2017) combines genome information with health care data from national registries. The project aims to collect data from 500,000 Finnish participants. Preterm and term birth were defined as births before and after 37 weeks of gestation. FinnGen preterm endpoint in Preparatory Phase Data Freeze 6 comprised individuals with World Health Organization International Classification of Diseases, Eight, Ninth, and Tenth Revision (ICD-8, ICD-9, and ICD-10) codes O60, 644, and 63497, respectively. We excluded births with ICD-9 code 644 (“early or threatened labor”) if they occurred after 37 weeks of gestation according to birth register data, and individuals with multiple gestation or birth, preeclampsia/eclampsia, and polyhydroamnios. Controls were people with spontaneous term birth. The GWAS of SPTB comprised 4,925 cases and 49,105 controls, and the GWAS of gestational duration comprised 24,391 mothers for whom gestational duration was available. We additionally performed GWAS with a “strict” definition of SPTB, in which we only included cases with births indicated as spontaneous and preterm in the endpoint data.

The study subjects from **Northern and Central Finland** were sampled in Oulu and Tampere University Hospital districts. SPTB was defined as birth prior to 36 wk + 1 d of gestation. Term birth was defined as birth at 38–41 wk (38 wk + 0 d to 41 wk + 6 d) of gestation. We excluded births with multiple gestation, preeclampsia, polyhydroamnios, intrauterine growth restriction, placental abruption, anomalies of the fetus, clinical chorioamnionitis or acute septic infection in the mother, alcohol or narcotic use, and accidents. Term births were from families without previous preterm births. The analysis comprised 286 cases and 488 controls.

Summary statistics of the **23andMe research program** were obtained by request. The summary data comprised unrelated mothers of European ancestry with self-reported gestational duration for their first singleton live birth. Individuals reporting a medical indication for preterm delivery were excluded. Preterm birth was defined as birth before 37 weeks of gestation, and control samples were people with term deliveries (>37 weeks). The meta-analysis of gestational duration included data from 43,567 individuals, and the meta-analysis of SPTB comprised 43,566 samples (3331 cases and 40,235 controls).

Data used in the replication and joint analysis originated from European women in the **Nordic data sets** including FIN cohort (N = 888; Finland), MoBa (N = 1,834; Norway), and DNBC (N = 5,921; Danish national birth cohort, Denmark), for which the summary statistics were obtained via collaboration. The characteristics of the data sets were described earlier [8, 41, 42]. Briefly, the samples from the Nordic cohorts were enriched for preterm births, and samples linked to births that were post-term or close to the preterm–term boundary of 37–38 weeks of gestation were excluded. The included preterm births were spontaneous, and births with obstetric induction of labor, preeclampsia, placental abnormalities, congenital malformations, and multiple births were excluded [41]. The mothers from the Finnish birth cohorts were collected for a genetic study of preterm birth [43] in Helsinki (southern Finland) University Hospital between 2004 and 2014. The mothers from MoBa originate from Norwegian Mother, Father and Child Cohort Study, for which pregnant women were recruited from 1998 to 2008, and the mothers from the DNBC were collected during 1997–2002 [8, 41, 42].

### DNA sample preparation, genotyping, imputation, and quality control

Various methods were used to extract DNA from the FinnGen samples. Genotyping was done with Illumina and Affymetrix arrays (Illumina Inc, San Diego, CA and Thermo Fisher Scientific, Santa Clara, CA). Sample quality control (QC) entailed excluding individuals of uncertain sex, non-Finnish ancestry, high missingness (>5%), and excess heterozygosity (±4SD). For genotype QC, variants with missingness >2%, minor allele count (MAC)<3, and deviation from Hardy–Weinberg equilibrium (HWE) (*p*<1×10^-6^) were excluded. Imputation was conducted against a Finnish population–specific SISuv3 reference with Beagle4.1 [44]. Variants with imputation info (INFO)<0.7 were excluded (https://github.com/FINNGEN/finngen-documentation).

DNA from Norhern/Central Finnish study was extracted with UltraClean Blood DNA Isolation Kit (MO BIO Laboratories, Inc., Carlsbad, CA), Puregene Blood Core Kit (Qiagen, Hilden, Germany), or prepIT-L2P kit (DNA Genotek, Ontario, Canada). Genotyping was performed with the Infinium HumanCoreExome BeadChip (Illumina, San Diego, CA) by the Technology Centre, Institute for Molecular Medicine Finland (FIMM), University of Helsinki. Variants with minor allele frequency (MAF)<1%, HWE *p*<1×10^-4^, or genotyping rate <90%, and samples with >10% missingness, were excluded. Prephasing was conducted with SHAPEIT2 [45], and imputation with IMPUTE2 [46], against the 1000 Genomes Project (1KGP) v3 reference panel [47]. Variants with INFO<0.7 were excluded.

DNA of the 23andMe samples was extracted from saliva samples, followed by genotyping with custom Illumina platforms by the National Genetics Institute (NGI). Samples with <97% European ancestry, and variants with HWE *p*<1×10^-20^, call rate <95%, or allele frequency discrepancy with 1KGP Europeans, were excluded. Imputation was done with Minimac242, using the 1KGP phase1 [47].

### GWAS and meta-analysis

FinnGen GWAS was conducted with Scalable and Accurate Implementation of Generalized mixed model (SAIGE) [48]. Gestational duration were inverse normalized. GWAS covariates were age, sex, genotyping batch, and the ten leading principal components. MAC was set to five. We used SNPTESTv2 [49] in GWAS of Northern/Central Finnish cohort. A frequentist case-control association test was implemented for SPTB, and a quantitative trait test for gestational duration was carried out with a linear model. Covariates were three multidimensional scaling (MDS) dimensions, defined with Plink1.9 [50]. We used SNPTEST defaults to achieve mean centering and scaling of gestational duration and to apply quantile normalization. Post-GWAS QC entailed excluding variants with MAF<1% and SNPTEST info<0.7. In the 23andMe data, preterm birth was analyzed with logistic regression, and linear regression was applied in the GWAS of gestational duration. Covariates were maternal age and the top five principal components.

We used METAL [51] to conduct a fixed-effect inverse varianc–weighted meta-analysis of SPTB and a sample size–weighted *p*-value-based meta-analysis of gestational duration. Sample size-based meta-analysis allows combining results when *β*-coefficients and standard errors from individual studies are in different units. Genomic coordinates of the meta-analysis cohorts were aligned into the GRCh38 coordinates. Genomic inflation factor was calculated with linkage disequilibrium score regression (LDSC) [12]. We report associations based on at least two individual meta-analysis cohorts, and excluded remaining rare variants with MAF<0.01%.

### Characterization of association signals

We defined associated loci as genomic regions within a ±1 Mb window around the lead variant. The locus was defined as novel if there were no previous genome-wide significant associations for SPTB or gestational duration in the ±1 Mb window in the National Human Genome Research Institute–European Bioinformatics Institute (NHGRI-EBI) GWAS Catalog or in the ±1 Mb window surrounding the loci reported in another recent meta-analysis of the timing of parturition, and no LD between the meta-analysis lead variants and previously reported index variants [9–11].

We used LDSC to estimate SNP-based heritability and to test for genetic correlation between gestational duration or SPTB with a comprehensive set of phenotypes downloaded from the Integrative Epidemiology Unit (IEU) OpenGWAS Project [12, 13]. We used FUMA GWAS (Functional Mapping and Annotation of Genome-Wide Association Studies) [52] to aid functional annotation of the GWAS results. FUMA was used to prioritize genes for enrichment testing and assessment and visualization of tissue-specific expression among GTExv8 tissues [53]. FUMA implements MAGMA (Multi-marker Analysis of GenoMic Annotation) [54] in gene-based analyses and gene-set enrichment analyses for GWAS summary data with curated gene sets and GO terms from Molecular Signature Database, MsigDB. In addition, we tested lists of gestational duration- and STPB-associated candidate genes for averaged gene expression across GTExv8 tissues, visualized in a heatmap with hierarchical clustering (Average/UPGMA[Unweighted Pair Group Method with Arithmetic Mean]) for genes and tissues, and for enrichment against various gene sets with hypergeometric tests in FUMA’s GENE2FUNC process.

To gain insight into the associated loci, we checked previous associations in the FinnGen R7 data and performed phenome-wide association study (PheWAS) within 1 Mb window around the meta-analysis index variants by querying GWAS data in the IEU OpenGWAS Project, which includes approximately 40,000 studies [13]. We performed colocalization analysis with HyprColoc [55] to assess if associated variants were also quantitative trait loci (QTLs) that affect mRNA expression. Colocalization was tested for variants within a 1Mb window around the meta-analysis lead variant. We did not iterate the runs for potential colocalization with further eGenes per tissue. We estimated betas from the meta-analysis Z-scores [56]. We used expression QTLs (eQTLs) with FDR<0.05 from the eQTLCatalogue [57], which contains uniformly processed cis-eQTLs from most of the available public studies. From the GTEx data in the eQTLCatalogue, we included eQTLs based on GTEx v8 and LCLs from an earlier release. We report colocalization results with a posterior probability (PP)>0.6 and meta-analysis *p*<5×10^-8^. We further investigated gene expression of the novel loci with evidence for colocalization in reproductive tissues in publicly available single cell data from the reproductive cell types in a human cell atlas of fetal development (https://descartes.brotmanbaty.org/bbi/human-gene-expression-during-development/) [22] and in reproductive cell atlas (https://www.reproductivecellatlas.org/) [20, 21]. We also checked the novel loci in the single cell data if the literature-indicated function of the gene was related to functionality in reproductive tissues during pregnancy.

### Replication and joint analysis

We tested the lead variants within each locus for association in the replication data from the data set of Nordic birth studies. For most of the tested variants, replication data from two out of three cohorts (FIN and MoBa, *n* = 2,722) was available. Joint test of the replication data and meta-analysis variants with *p*<1×10^-6^ was done to see if additional loci reached genome-wide significance. We did not test replicated loci originally reported in Zhang et al. [8] since the same replication data was used.

## Supporting information

S1 Fig Overview of the study data sets in the GWAS and meta-analysis populations, and in replication. (PDF)

S2 Fig Meta-analysis of gestational duration (upper panel) and SPTB in which a strict definition of spontaneous birth was used in the GWAS of the FinnGen data. The meta-analysis of gestational duration was performed with 66,001 samples whereas the meta-analysis of SPTB included 94,781 samples (4,953 cases and 89,828 controls).
(PDF)

S3 Fig Effect estimates for the genome-wide significant meta-analysis loci in the FinnGen based GWAS. The genes mapped to the meta-analysis risk loci for gestational duration and SPTB are shown in alphabetical order. Effect estimates for *EBF1* and *EEFSEC* are from the GWAS of gestational duration. The main GWAS of gestational duration was performed with data from 24,391 samples, and the GWAS with strict definition with 21,660 samples. The main GWAS of SPTB was based on 54,030 samples (4,925 cases and 49,105 controls), whereas the strict GWAS of SPTB had 50,441 samples (1,336 cases and 49,105 controls). The variants with a minor allele count (MAC) of five shown.
(PDF)

S4 Fig QQ-plots of the meta-analysis of gestational duration (on the left) and SPTB (on the right). (PDF)

S5 Fig Effect estimates of the genome-wide significant loci from the meta-analysis populations of SPTB. Two variants in *EBF1* and *EEFSEC* loci are shown because the meta-analysis lead variants were not present in the FinnGen-based GWAS.
(PDF)

S6 Fig Gene expression of gestational duration- and SPTB-annotated genes across 30 tissue types in GTEx v8. The histograms show MAGMA gene set enrichment analysis for gestational duration (above) and SPTB (below). The heatmap features gene expression depicted as averaged expression value per tissue type, with hierarchal clustering for both genes and tissues. Across genes and tissues, the cells filled in red represent higher expression compared to the cells filled in blue.
(PDF)

S7 Fig Regional association plots of the novel loci associated with gestational duration. (PDF)

S8 Fig Regional association plots of the novel loci associated with SPTB. (PDF)

S9 Fig Associations of the candidate genes from the meta-analysis of SPTB and gestational duration in the FinnGen R7 GWAS endpoint categories, each comprising >3,000 traits. In each category, *p* value is based on the strongest associating trait.
(PDF)

S1 Table Properties of the phenotype and genotype data and data handling of the meta-analysis cohorts. (PDF)

S2 Table Phenotype data of the meta-analysis cohorts. (XLSX)

S3 Table Gestational duration- and SPTB-associated loci without previous associations (± 1Mb around the meta-analysis lead variant) with preterm birth in the GWAS Catalog. (XLSX)

S4 Table LDSC based SNP heritability of gestational duration and SPTB in the FinnGen GWAS and in the meta-analysis. (PDF)

S5 Table LDSC based genetic correlation among gestational duration (GD) or SPTB and a set of 773 other complex traits. (XLSX)

S6 Table MAGMA gene set enrichment analysis. Gene sets with adjusted *p*<0.05 or top 10 gene sets shown.
(PDF)

S7 Table Replication and joint analysis of associated lead variants with *p*<5e^-8^ and *p*<1e^-6^. Loci near *WNT4, EEFSEC, ADCY5, EBF1*, and *AGTR2*, originally discovered in NEJM 21;377:1156–1167 were not included, since the same replication data set was used in that study.
(XLSX)

S8 Table Gene set enrichment analysis (FUMA Gene2Func) for genes assigned into the associated (*p*<5e-8) loci in the meta-analysis of gestational duration and SPTB, and in the joint analysis with the replication data. (XLSX)

S9 Table PheWAS of the genome-wide significant variants in the meta-analysis loci ±1Mb around the index variant. (XLSX)

S10 Table Colocalization analysis of the associated meta-analysis loci with 1Mb window around the lead variant. Results with PP>0.6 and meta-analysis *p*<1e-6 shown.
(XLSX)

S1 Acknowledgements Contributors of FinnGen. (XLSX)
