# Supplementary material for: Meta-analysis of genome-wide association studies of gestational duration and spontaneous preterm birth identifies new maternal risk loci
Source: PLoS Genet. 2023 Oct 23;19(10):e1010982. doi: 10.1371/journal.pgen.1010982 (PMC10621942; doi:10.1371/journal.pgen.1010982)
Supplement: S1 Fig — (PDF) [file pgen.1010982.s001.pdf]

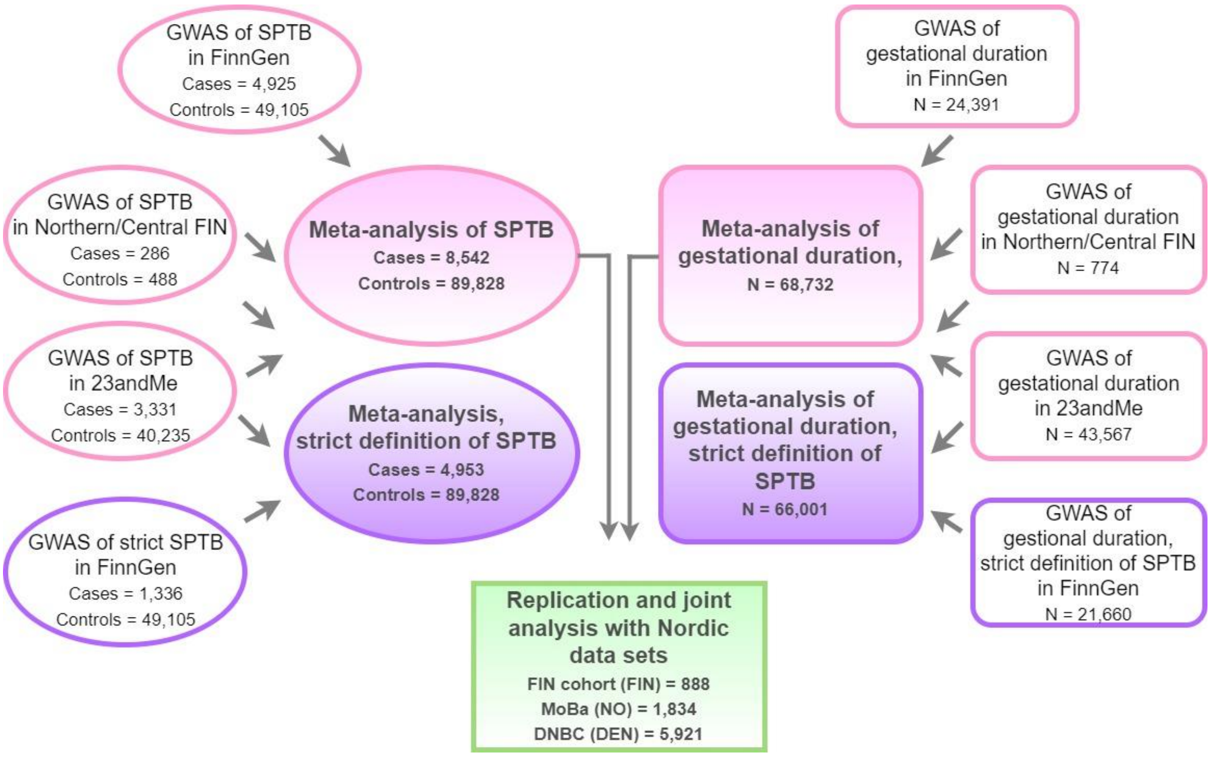

S1 Fig. Overview of the study data sets in the GWAS and meta-analysis populations, and in replication.
