# Supplementary material for: Meta-analysis of genome-wide association studies of gestational duration and spontaneous preterm birth identifies new maternal risk loci
Source: PLoS Genet. 2023 Oct 23;19(10):e1010982. doi: 10.1371/journal.pgen.1010982 (PMC10621942; doi:10.1371/journal.pgen.1010982)
Supplement: S2 Fig — The meta-analysis of gestational duration was performed with 66,001 samples whereas the meta-analysis of SPTB included 94,781 samples (4,953 cases and 89,828 controls). (PDF) [file pgen.1010982.s002.pdf]

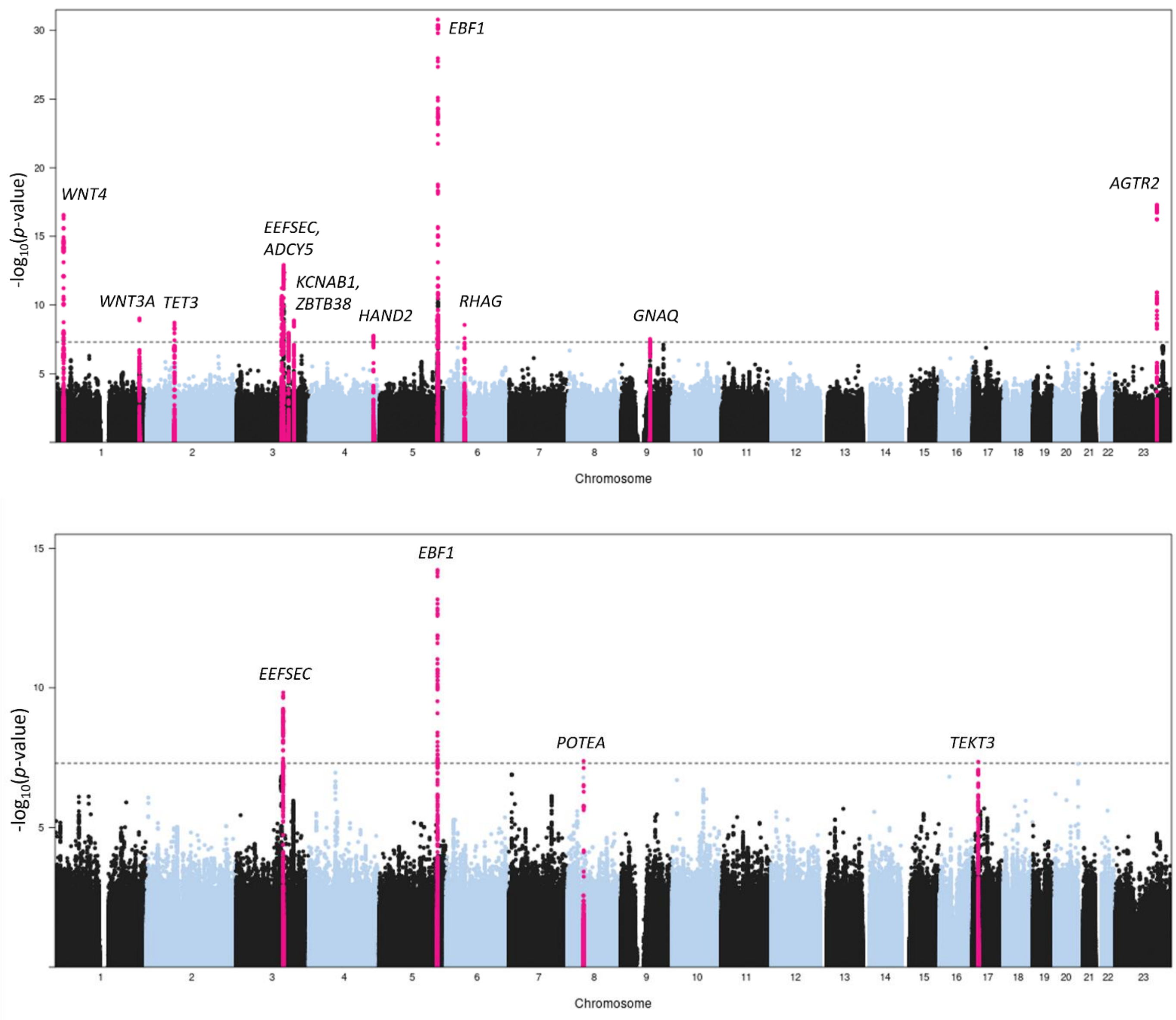

S2 Fig. Meta-analysis of gestational duration (upper panel) and SPTB in which a strict definition of spontaneous birth was used in the GWAS of the FinnGen data. The meta-analysis of gestational duration was performed with 66,001 samples whereas the meta-analysis of SPTB included 94,781 samples (4,953 cases and 89,828 controls).
