# Supplementary material for: Meta-analysis of genome-wide association studies of gestational duration and spontaneous preterm birth identifies new maternal risk loci
Source: PLoS Genet. 2023 Oct 23;19(10):e1010982. doi: 10.1371/journal.pgen.1010982 (PMC10621942; doi:10.1371/journal.pgen.1010982)
Supplement: S3 Fig — The genes mapped to the meta-analysis risk loci for gestational duration and SPTB are shown in alphabetical order. Effect estimates for EBF1 and EEFSEC are from the GWAS of gestational duration. The main GWAS of gestational duration was performed with data from 24,391 samples, and the GWAS with strict definition with 21,660 samples. The main GWAS of SPTB was based on 54,030 samples (4,925 cases and 49,105 controls), whereas the strict GWAS of SPTB had 50,441 samples (1,336 cases and 49,105 controls). The variants with a minor allele count (MAC) of five shown. (PDF) [file pgen.1010982.s003.pdf]

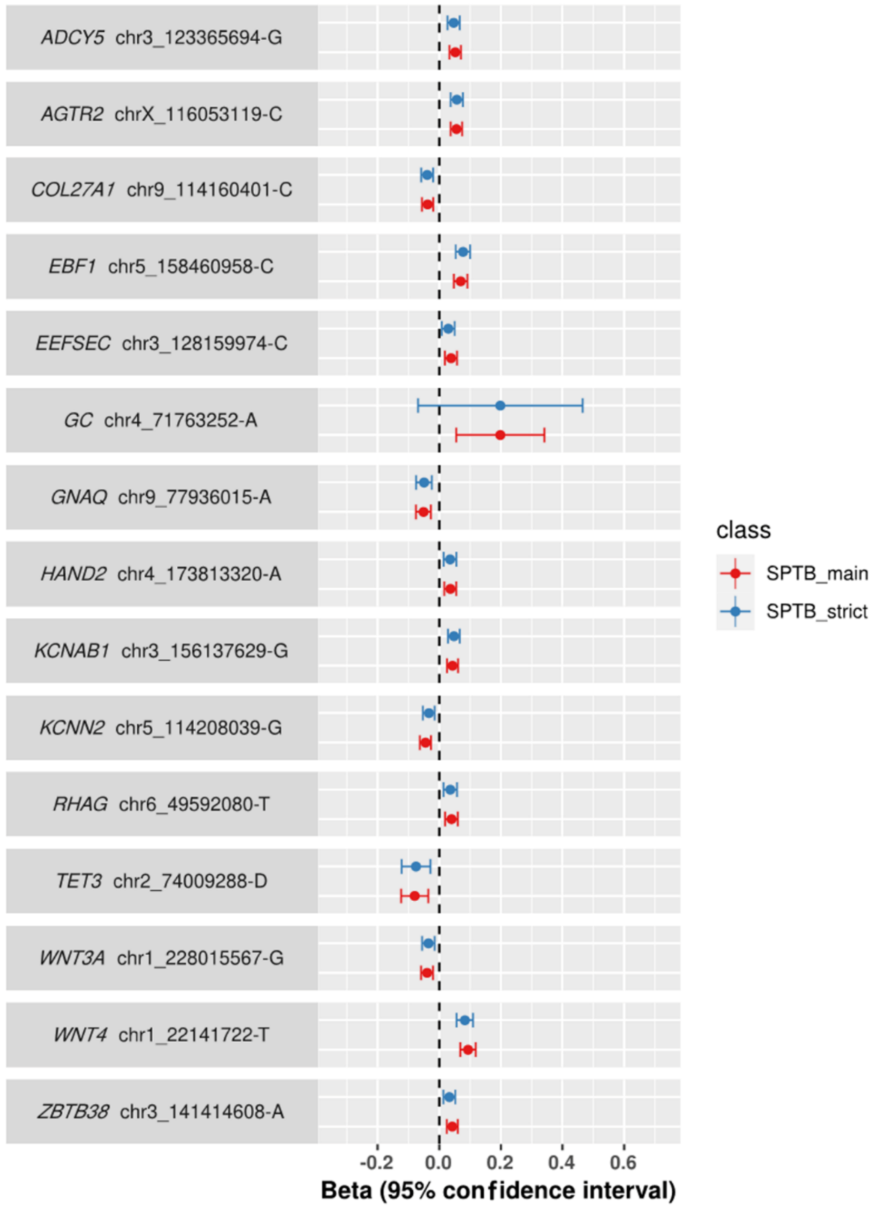

S3 Fig. Effect estimates for the genome-wide significant meta-analysis loci in the FinnGen based GWAS. The genes mapped to the meta-analysis risk loci for gestational duration and SPTB are shown in alphabetical order. Effect estimates for *EBF1* and *EEFSEC* are from the GWAS of gestational duration. The main GWAS of gestational duration was performed with data from 24,391 samples, and the GWAS with strict definition with 21,660 samples. The main GWAS of SPTB was based on 54,030 samples (4,925 cases and 49,105 controls), whereas the strict GWAS of SPTB had 50,441 samples (1,336 cases and 49,105 controls). The variants with a minor allele count (MAC) of five shown.
