# Supplementary figures and images for: Meta-analysis of genome-wide association studies of gestational duration and spontaneous preterm birth identifies new maternal risk loci
Source: PLoS Genet. 2023 Oct 23;19(10):e1010982. doi: 10.1371/journal.pgen.1010982 (PMC10621942; doi:10.1371/journal.pgen.1010982)

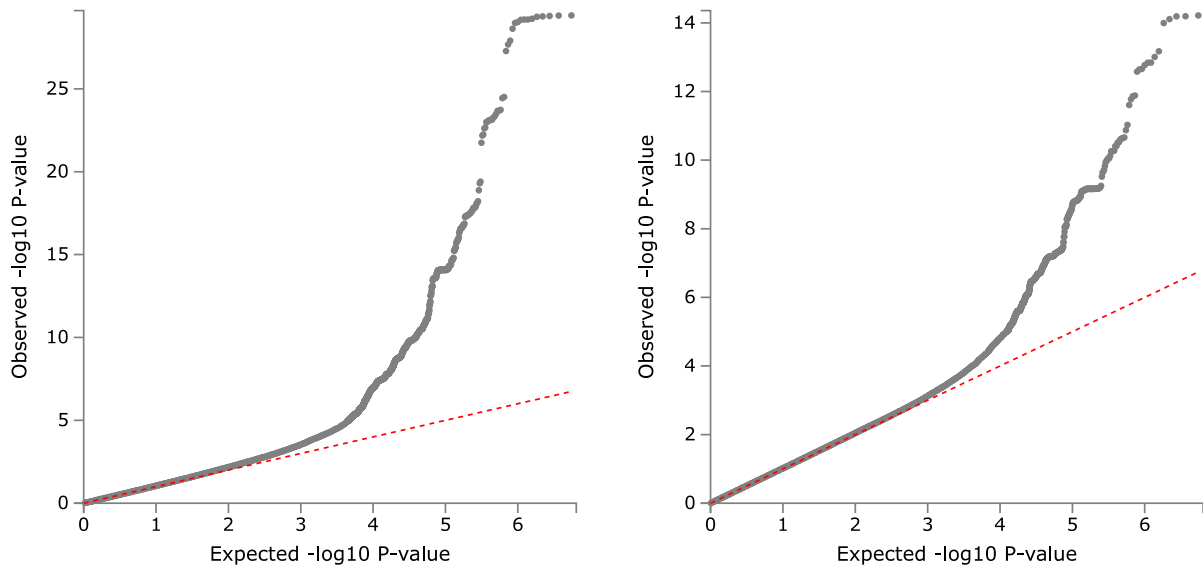

**S4 Fig. QQ-plots of the meta-analysis of gestational duration (on the left) and SPTB (on the right).**

Supplement: S4 Fig — (PDF) [file pgen.1010982.s004.pdf]

**S7 Fig. Regional association plots of the novel loci associated with gestational duration.**

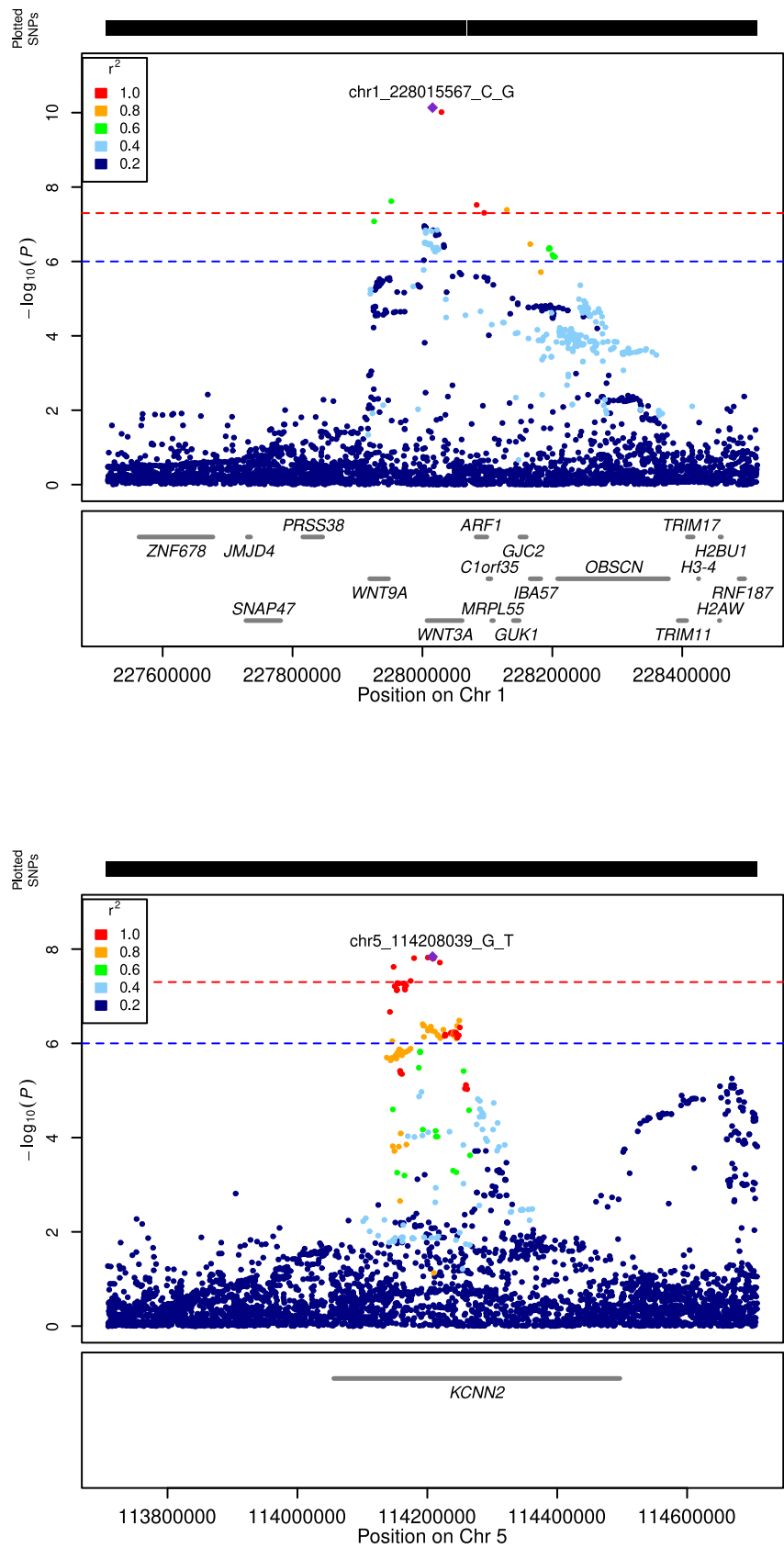

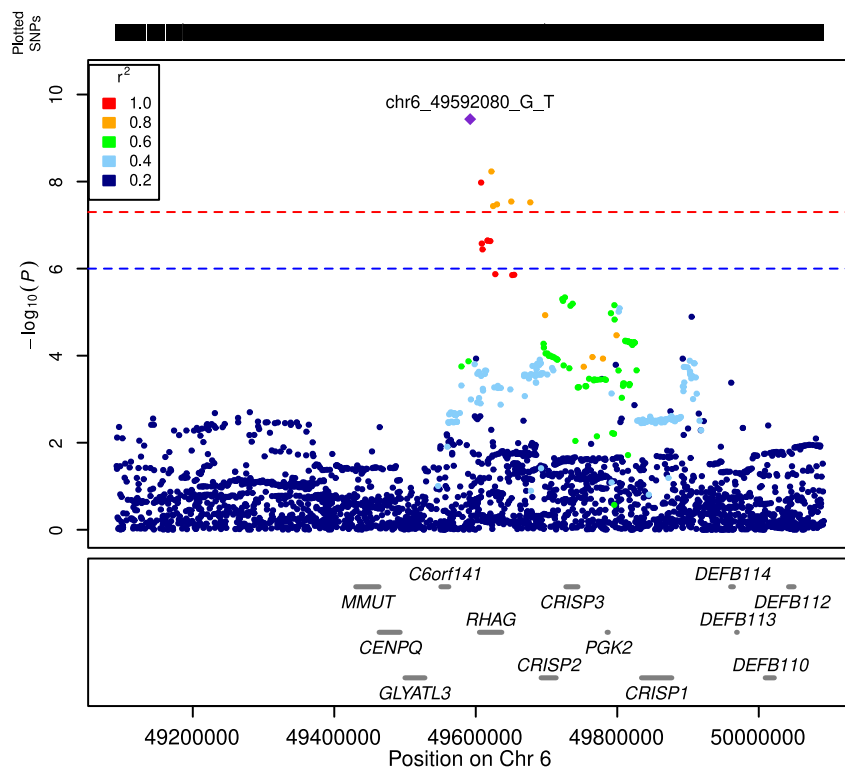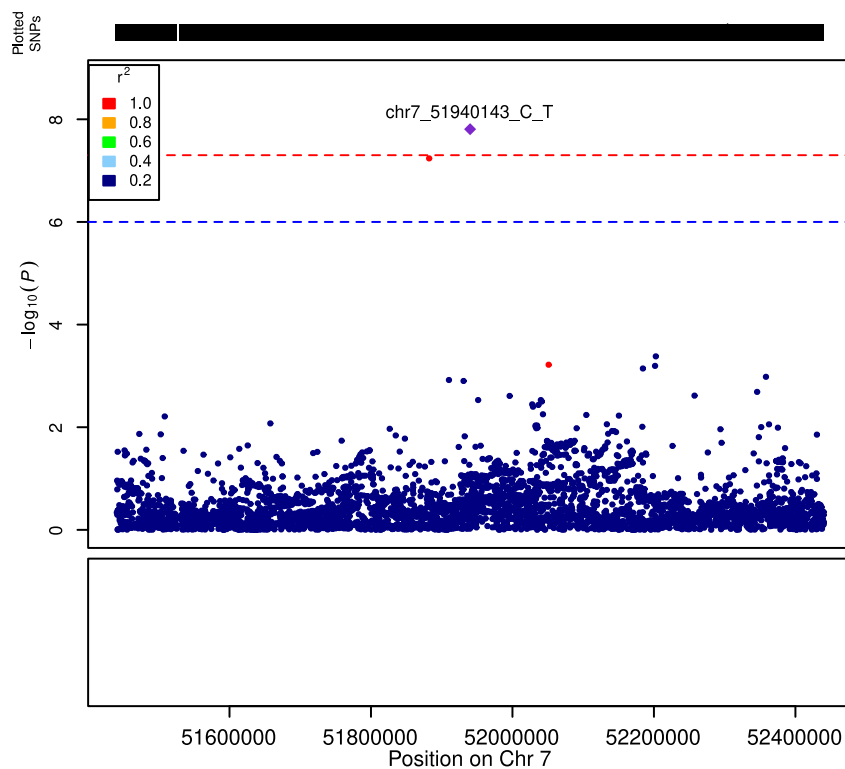

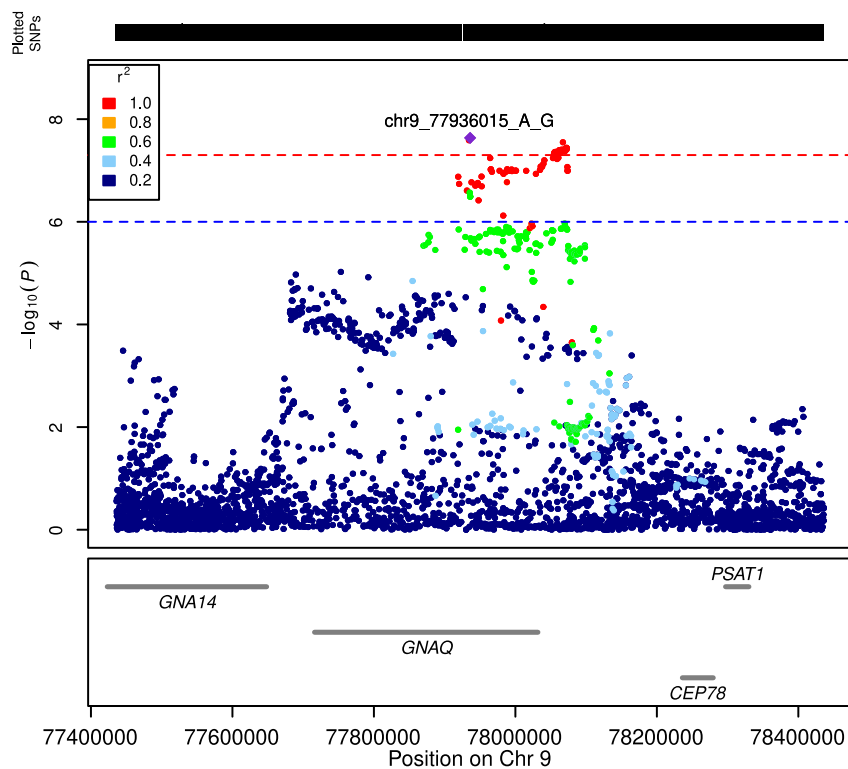

Supplement: S7 Fig — (PDF) [file pgen.1010982.s007.pdf]

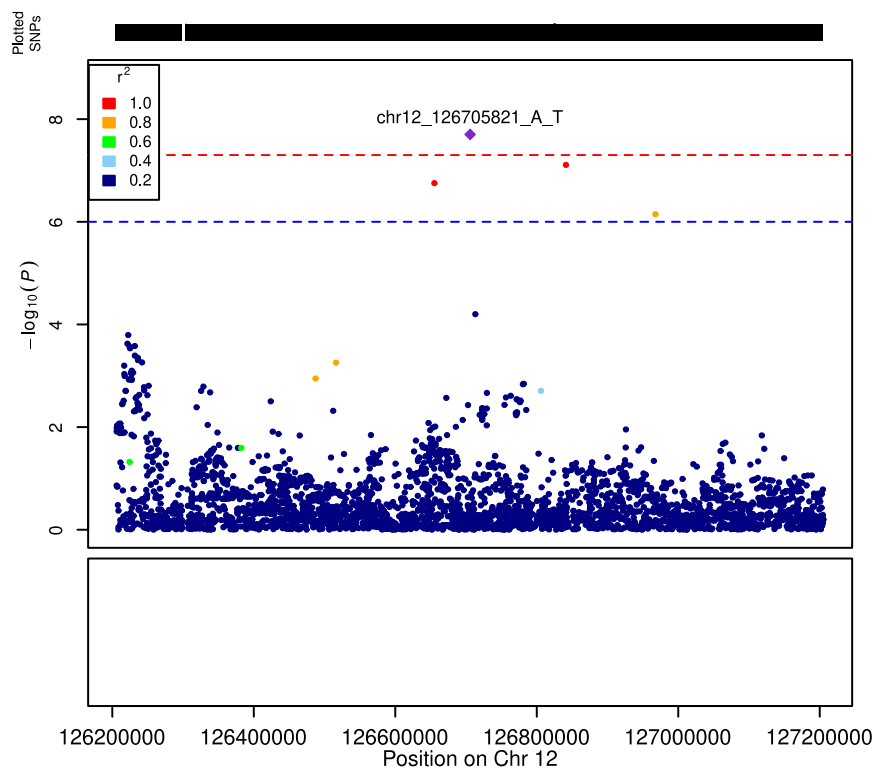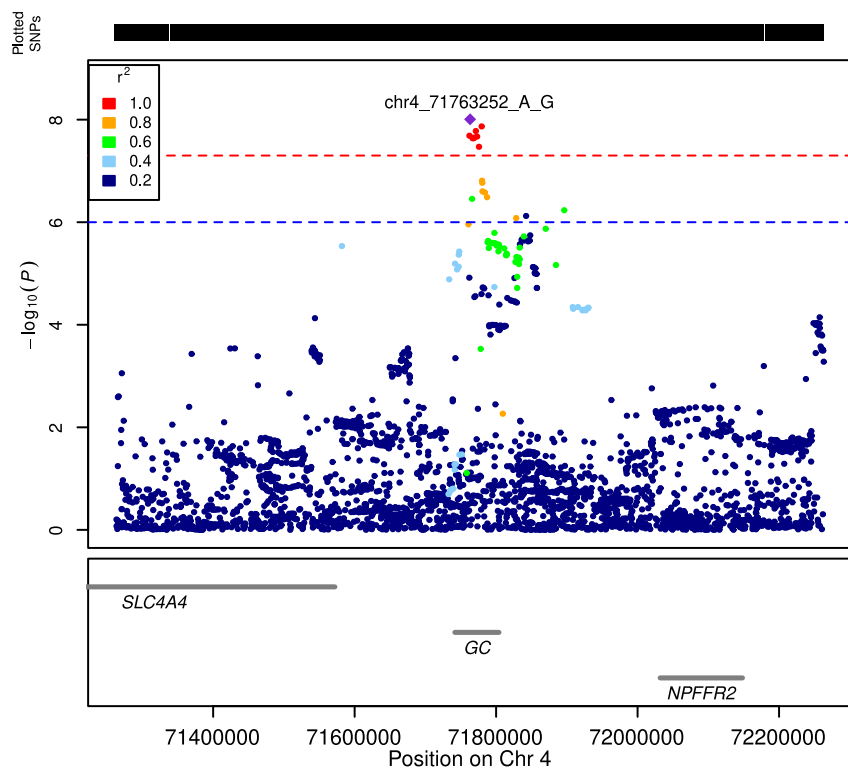

**S8 Fig. Regional association plots of the novel loci associated with SPTB.**

Supplement: S8 Fig — (PDF) [file pgen.1010982.s008.pdf]
