# Supplementary material for: Meta-analysis of genome-wide association studies of gestational duration and spontaneous preterm birth identifies new maternal risk loci
Source: PLoS Genet. 2023 Oct 23;19(10):e1010982. doi: 10.1371/journal.pgen.1010982 (PMC10621942; doi:10.1371/journal.pgen.1010982)
Supplement: S5 Fig — Two variants in EBF1 and EEFSEC loci are shown because the meta-analysis lead variants were not present in the FinnGen-based GWAS. (PDF) [file pgen.1010982.s005.pdf]

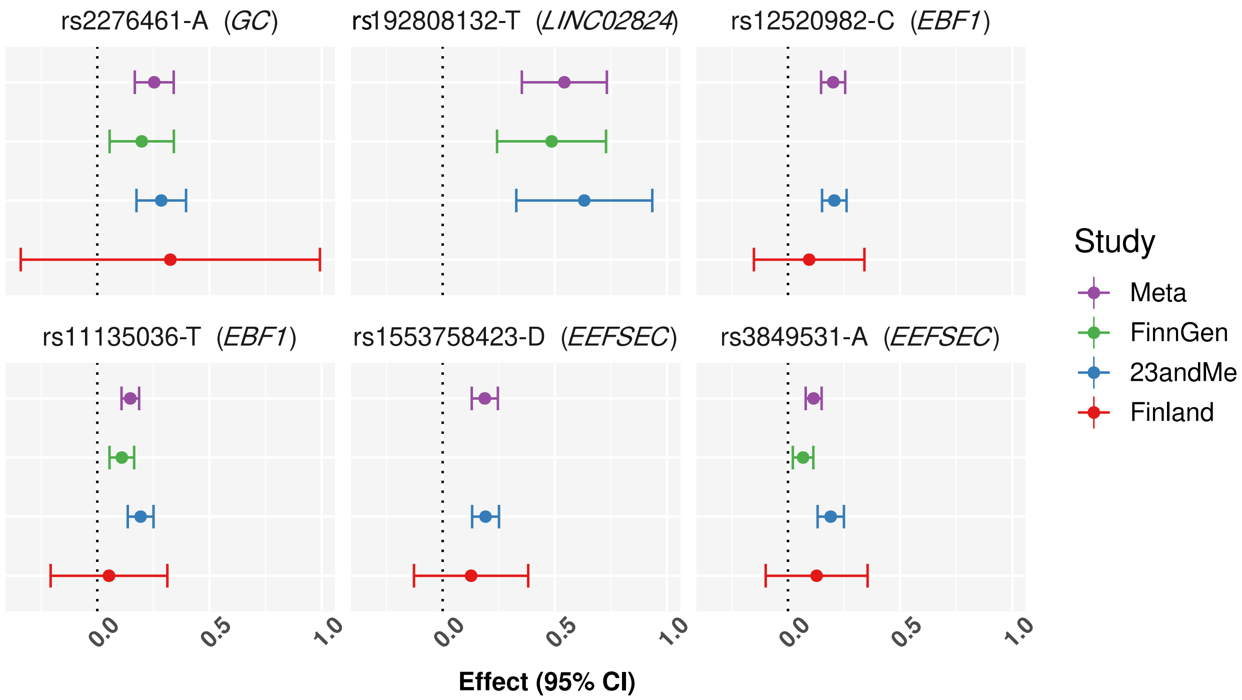

S5 Fig. **Effect estimates of the genome-wide significant loci from the meta-analysis populations of SPTB.** Two variants in *EBF1* and *EEFSEC* loci are shown because the meta-analysis lead variants were not present in the FinnGen-based GWAS.
