# Supplementary material for: Meta-analysis of genome-wide association studies of gestational duration and spontaneous preterm birth identifies new maternal risk loci
Source: PLoS Genet. 2023 Oct 23;19(10):e1010982. doi: 10.1371/journal.pgen.1010982 (PMC10621942; doi:10.1371/journal.pgen.1010982)
Supplement: S6 Fig — The histograms show MAGMA gene set enrichment analysis for gestational duration (above) and SPTB (below). The heatmap features gene expression depicted as averaged expression value per tissue type, with hierarchal clustering for both genes and tissues. Across genes and tissues, the cells filled in red represent higher expression compared to the cells filled in blue. (PDF) [file pgen.1010982.s006.pdf]

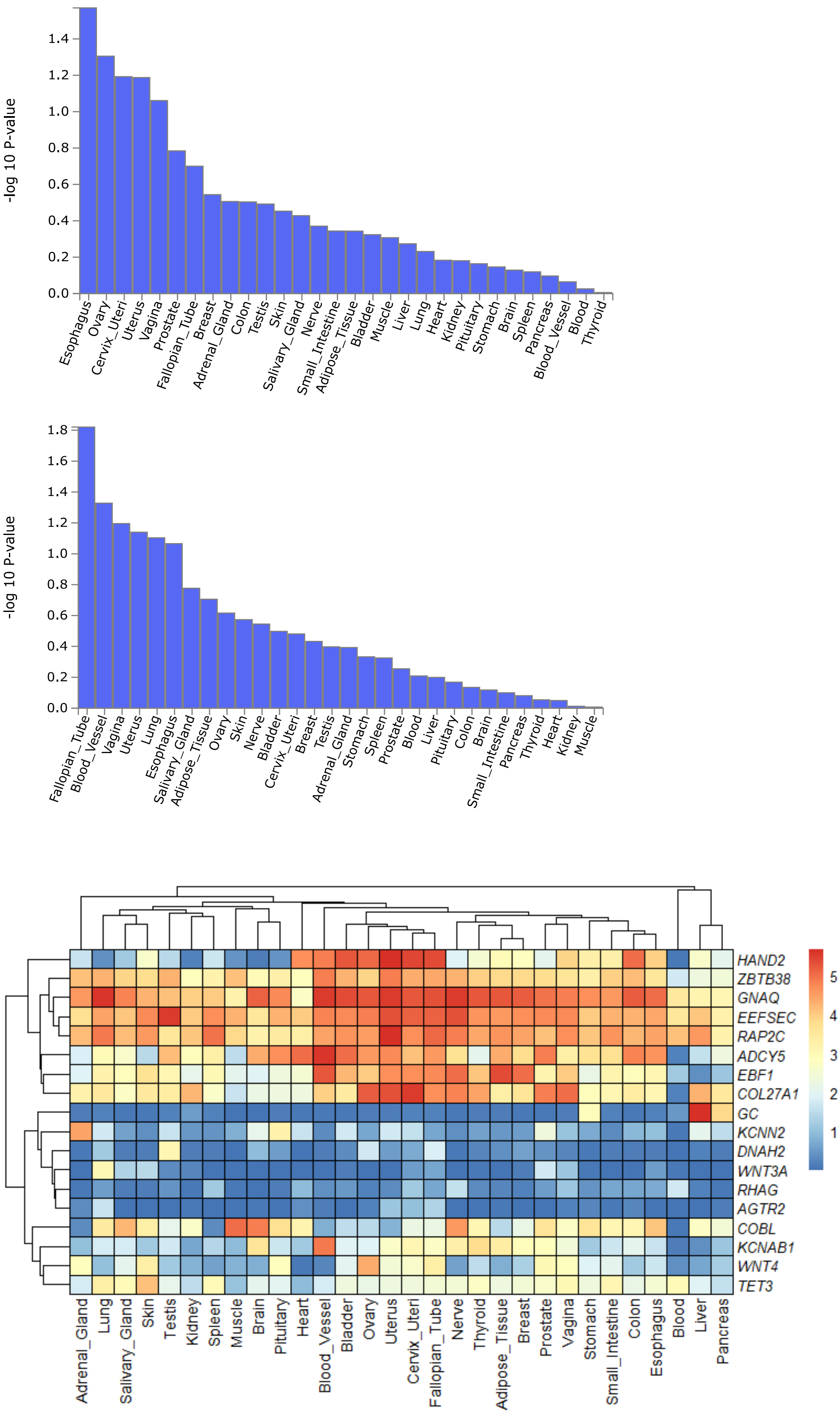

S6 Fig. **Gene expression of gestational duration- and SPTB-annotated genes across 30 tissue types in GTEx v8**. The histograms show MAGMA gene set enrichment analysis for gestational duration (above) and SPTB (below).The heatmap features gene expression depicted as averaged expression value per tissue type, with hierarchal clustering for both genes and tissues. Across genes and tissues, the cells filled in red represent higher expression compared to the cells filled in blue.
