# Supplementary material for: Meta-analysis of genome-wide association studies of gestational duration and spontaneous preterm birth identifies new maternal risk loci
Source: PLoS Genet. 2023 Oct 23;19(10):e1010982. doi: 10.1371/journal.pgen.1010982 (PMC10621942; doi:10.1371/journal.pgen.1010982)
Supplement: S9 Fig — In each category, p value is based on the strongest associating trait. (PDF) [file pgen.1010982.s009.pdf]

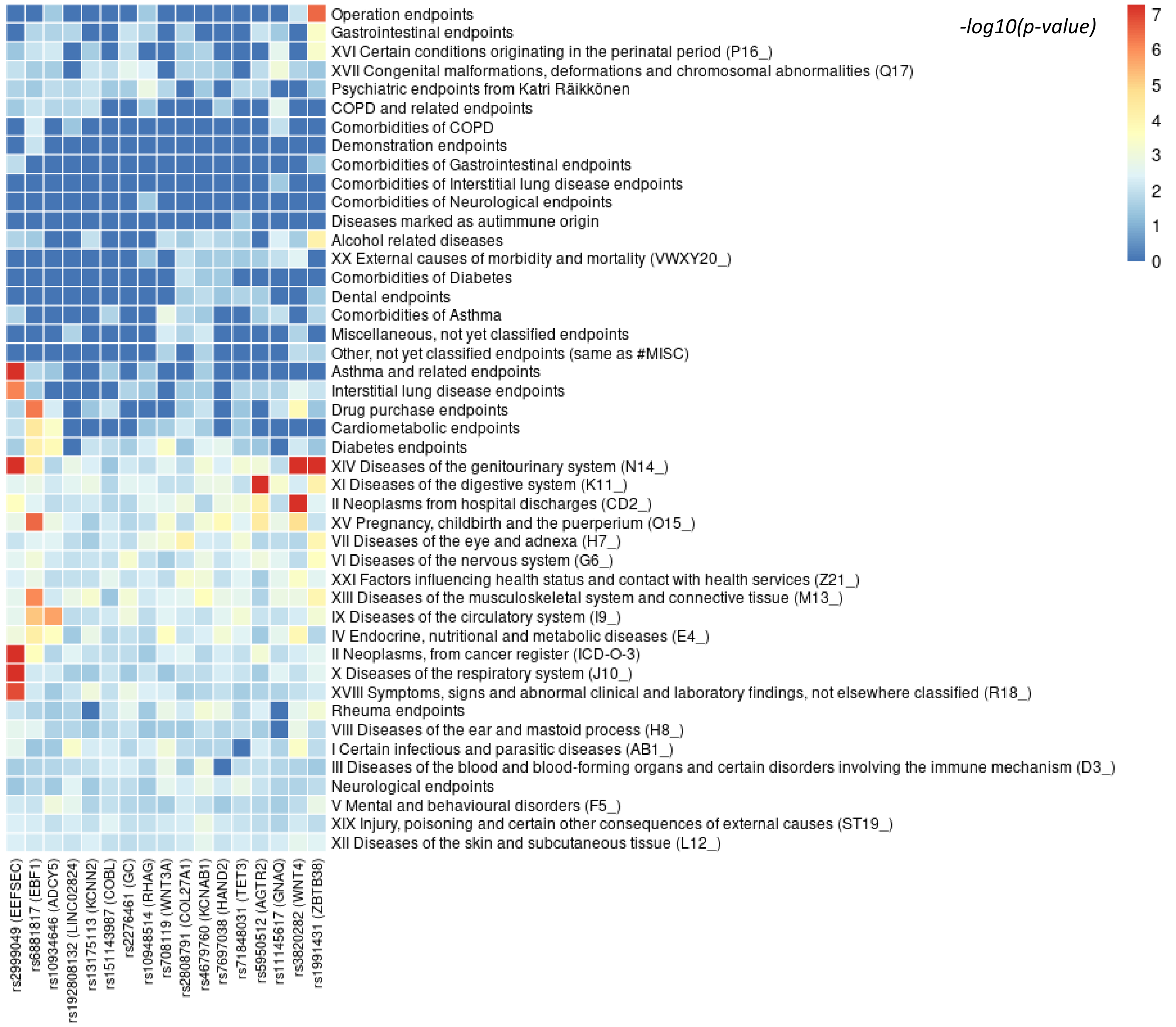

S9 Fig. Associations of the candidate genes from the meta-analysis of SPTB and gestational duration in the FinnGen R7 GWAS endpoint categories, each comprising >3,000 traits. In each category, p value is based on the strongest associating trait.
