# Supplementary material for: Meta-analysis of genome-wide association studies of gestational duration and spontaneous preterm birth identifies new maternal risk loci
Source: PLoS Genet. 2023 Oct 23;19(10):e1010982. doi: 10.1371/journal.pgen.1010982 (PMC10621942; doi:10.1371/journal.pgen.1010982)
Supplement: S1 Table — (PDF) [file pgen.1010982.s010.pdf]

S1 Table. Properties of the phenotype and genotype data and data handling of the meta-analysis cohorts.

	Phenotype data			Genotype data				
	**Source**	**Definition of preterm birth**	**Exclusion criteria**	**Quality control prior to GWAS**	**Data tranformation**	**GWAS Method**	**GWAS covariates**	**Quality control after GWAS**
FinnGenR6	Register-based	Based on ICD-codes and gestational duration. ICD8, 63497 (Partus prematurus imminens, alibi non classificabilis, at 29-37 weeks of gestation); ICD9, 644A (partus praematurus imminensis), individuals with term births (>37wk) excluded; ICD9, 644B (partus praematurus, at 29-37 weeks of gestation); ICD10, O60 (preterm birth, delivery started <37 weeks of gestation)	Multiple gestation or birth, pre- or eclampsia, polyhydroamnios	Sample exclusion criteria: ambiguous sex, non-Finnish ancestry, missingness >5%, excess heterozygosity (+/-4SD); Variant exclusion criteria: missingness >2%, minor allele count (MAC)<3, deviation from HWE ($p < 1\times10^{-6}$), imputation INFO <0.7	Gestational duration were inverse-normalized	Linear / logistic regression with Scalable and Accurate Implementation of Generalized mixed model, SAIGE, MAC=5	Maternal age, genotyping batch, the 10 leading principal components	Variant exclusions: MAF<0.01%
Northern/Central Finland	Register-based	Based on gestational duration: Preterm, <36 wk + 1d of gestation; term > 38–41 wk + 6 d of gestation	Multiple gestation or birth, pre- or eclampsia, polyhydroamnios, intrauterine growth restriction, placental abruption, anomalies of the fetus, clinical chorioamnionitis or acute septic infection in the mother, alcohol or narcotic use, accidents	Sample exclusion criteria: high missingness (>10%), uncertain sex; Variant exclusion criteria: minor allele frequency (MAF)<1%, deviation from HWE ($p < 1\times10^{-4}$), genotyping rate <90%, imputation INFO <0.7	Gestational duration were mean centered, scaled, and quantile normalized	Frequentist association test / quantitative trait test with SNPTESTv2	The top three MDS (multidimensional scaling) components	Variant exclusions: MAF<1%, SNPTEST info<0.7
23andMe	Self-reported	Based on gestational duration: Preterm, <37 wk of gestation; term >37wk of gestation	Medical indication for preterm delivery	Sample exclusion criteria: <97% of European ancestry; Variant exclusion criteria: deviation from HWE ($p < 1\times10^{-20}$), call rate <95%, allele frequency discrepancy with 1000Genomes Europeans	None	Linear / logistic regression	Maternal age and the top five principal components	Variant exclusions: MAF<0.01%
