# Supplementary material for: Meta-analysis of genome-wide association studies of gestational duration and spontaneous preterm birth identifies new maternal risk loci
Source: PLoS Genet. 2023 Oct 23;19(10):e1010982. doi: 10.1371/journal.pgen.1010982 (PMC10621942; doi:10.1371/journal.pgen.1010982)
Supplement: S4 Table — (PDF) [file pgen.1010982.s013.pdf]

S4 Table. LDSC based SNP heritability of gestational duration and SPTB in the FinnGen GWAS and in the meta-analysis.

	Gestational duration				
**population**	N_preterm	N_term	N_total	sample_prevalence	H^2 (se)
FinnGenR6*	2250	22141	24391	0,09224714	**0.1263 (0.0593)**
Finland	286	488	774	0,369509044	
23andMe	3331	40237	43568	0,076455196	
Meta-analysis	5867	62866	68733	0,085359289	**0.1747 (0.0266)**

	SPTB Case-Control				
**population**	N_preterm	N_term	N_total	sample_prevalence	H^2 (se)
FinnGenR6*	4925	49105	54030	0,091153063	**0.0181 (0.0248)**
Finland	286	488	774	0,369509044	
23andMe	3331	40236	43567	0,076456951	
Meta-analysis	8542	89829	98371	0,086834535	**0.0565 (0.0141)**
