# Supplementary material for: Meta-analysis of genome-wide association studies of gestational duration and spontaneous preterm birth identifies new maternal risk loci
Source: PLoS Genet. 2023 Oct 23;19(10):e1010982. doi: 10.1371/journal.pgen.1010982 (PMC10621942; doi:10.1371/journal.pgen.1010982)
Supplement: S6 Table — Gene sets with adjusted p<0.05 or top 10 gene sets shown. (PDF) [file pgen.1010982.s015.pdf]

**S6 Table. MAGMA gene set enrichment analysis.** Gene sets with adjusted *p* <0.05 or top 10 gene sets shown.

| Gene Set | N genes | Beta | Beta STD | SE | P | Pbon |
|---|---|---|---|---|---|---|
| **1) Gestational duration** | | | | | | |
| GO_bp:go_regulation_of_male_gonad_development | 8 | 1.6697 | 0.033679 | 0.32374 | 1.2642e-07 | 0.001958 |
| GO_bp:go_negative_regulation_of_gonad_development | 6 | 1.7937 | 0.031335 | 0.35575 | 2.3236e-07 | 0.003599 |
| GO_bp:go_regulation_of_steroid_hormone_biosynthetic_process | 10 | 1.2403 | 0.027969 | 0.25691 | 6.9596e-07 | 0.010778 |
| GO_bp:go_kidney_mesenchyme_development | 19 | 0.8523 | 0.026487 | 0.19948 | 9.6990e-06 | 0.150189 |
| Curated_gene_sets:naba_basement_membranes | 40 | 0.6294 | 0.028362 | 0.15166 | 1.6717e-05 | 0.258846 |
| GO_bp:go_mesenchymal_cell_differentiation_involved_in_kidney_development | 8 | 1.1576 | 0.023350 | 0.28038 | 1.8309e-05 | 0.283478 |
| GO_bp:go_regulation_of_glucocorticoid_biosynthetic_process | 7 | 1.2095 | 0.022821 | 0.29547 | 2.1344e-05 | 0.330448 |
| GO_bp:go_primary_alcohol_biosynthetic_process | 11 | 0.9794 | 0.023162 | 0.25015 | 4.5343e-05 | 0.701955 |
| GO_bp:go_negative_regulation_of_ion_transport | 141 | 0.2822 | 0.023813 | 0.07226 | 4.7241e-05 | 0.731291 |
| GO_bp:go_regulation_of_aldosterone_metabolic_process | 7 | 1.2520 | 0.023623 | 0.32220 | 5.1185e-05 | 0.792293 |
| **2) SPTB** | | | | | | |
| GO_cc:go_kinetochore_microtubule | 7 | 0.9345 | 0.017629 | 0.26517 | 0.00021371 | 1 |
| GO_bp:go_neuron_differentiation | 1329 | 0.0843 | 0.021160 | 0.02393 | 0.00021529 | 1 |
| GO_bp:go_regulation_of_delayed_rectifier_potassium_channel_activity | 18 | 0.7170 | 0.021688 | 0.20799 | 0.00028397 | 1 |
| Curated_gene_sets:hoshida_liver_cancer_subclass_s1 | 236 | 0.1804 | 0.019653 | 0.05262 | 0.00030361 | 1 |
| GO_bp:go_actin_polymerization_dependent_cell_motility | 6 | 1.2503 | 0.021843 | 0.36631 | 0.00032184 | 1 |
| GO_bp:go_neurogenesis | 1573 | 0.0747 | 0.020276 | 0.02203 | 0.00034715 | 1 |
| Curated_gene_sets:berenjeno_transformed_by_rhoa_reversibly_dn | 30 | 0.5587 | 0.021814 | 0.16765 | 0.00043084 | 1 |
| GO_bp:go_cell_cell_adhesion_via_plasma_membrane_adhesion_molecules | 268 | 0.1985 | 0.023024 | 0.05994 | 0.00046403 | 1 |
| GO_bp:go_g_protein_coupled_purinergic_receptor_signaling_pathway | 22 | 0.6551 | 0.021907 | 0.19785 | 0.00046538 | 1 |
| Curated_gene_sets:mohankumar_hoxa1_targets_dn | 161 | 0.2111 | 0.019030 | 0.06392 | 0.00047957 | 1 |
